# Unraveling CoNiP‒CoP_2_ 3D‐on‐1D Hybrid Nanoarchitecture for Long‐Lasting Electrochemical Hybrid Cells and Oxygen Evolution Reaction

**DOI:** 10.1002/advs.202104877

**Published:** 2022-01-22

**Authors:** S. Chandra Sekhar, Bhimanaboina Ramulu, Man Ho Han, Shaik Junied Arbaz, Manchi Nagaraju, Hyung‐Suk Oh, Jae Su Yu

**Affiliations:** ^1^ Department of Electronics and Information Convergence Engineering, Institute for Wearable Convergence Electronics Kyung Hee University 1732 Deogyeong‐daero, Gihung‐gu Yongin‐si Gyeonggi‐do 17104 Republic of Korea; ^2^ Clean Energy Research Center Korea Institute of Science and Technology (KIST) Hwarang‐ro 14‐gil 5, Seongbuk‐gu Seoul 02792 Republic of Korea; ^3^ KHU‐KIST Department of Conversing Science and Technology Kyung Hee University Seoul 02447 Republic of Korea

**Keywords:** cashew fruit‐like nanostructures, CoNiP‒CoP_2_, hybrid cell, OER, operando XANES

## Abstract

Evolving cost‐effective transition metal phosphides (TMPs) using general approaches for energy storage is pivotal but challenging. Besides, the absence of noble metals and high electrocatalytic activity of TMPs allow their applicability as catalysts in oxygen evolution reaction (OER). Herein, CoNiP‒CoP_2_ (CNP‒CP) composite is in situ deposited on carbon fabric by a one‐step hydrothermal technique. The CNP‒CP reveals hybrid nanoarchitecture (3D‐on‐1D HNA), i.e., cashew fruit‐like nanostructures and nanocones. The CNP‒CP HNA electrode delivers higher areal capacity (82.8 μAh cm^–2^) than the other electrodes. Furthermore, a hybrid cell assembled with CNP‒CP HNA shows maximum energy and power densities of 31 μWh cm^–2^ and 10.9 mW cm^–2^, respectively. Exclusively, the hybrid cell demonstrates remarkable durability over 30 000 cycles. In situ/operando X‐ray absorption near‐edge structure analysis confirms the reversible changes in valency of Co and Ni elements in CNP‒CP material during real‐time electrochemical reactions.  Besides, a quasi‐solid‐state device unveils its practicability by powering electronic components. Meanwhile, the CNP‒CP HNA verifies its higher OER activity than the other catalysts by revealing lower overpotential (230 mV). Also, it exhibits relatively small Tafel slope (38 mV dec^–1^) and stable OER activity over 24 h. This preparation strategy may initiate the design of advanced TMP‐based materials for multifunctional applications.

## Graphical abstract

CoNiP‒CoP_2_ (CNP‒CP) hybrid nanoarchitecture (HNA) is in situ deposited on flexible carbon fabric via a facile one‐step hydrothermal technique, which exhibits cashew fruit‐like nanostructures and nanocones. Exploiting multifunctional aspects, the CNP‒CP HNA serves as a potential bifunctional material for hybrid cells as well as oxygen evolution reactions.

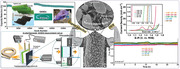

## 1 Introduction

The thriving of wearable and portable electronic gadgets in recent years has stimulated the quest for pertinent flexible and long‐lasting power devices.^[^ ^1^ ^]^ Supercapacitors (SCs) and lithium‐ion batteries (LIBs) take a lead to power these portable electronics among other energy storage systems. Specifically, SCs have attracted increasing interest over LIBs in terms of safety, durability, power density, and even flexibility.^[^ ^2^ ^]^ SCs in a hybrid cell configuration with high redox‐active battery‐type materials and porous carbon‐related materials as positive and negative electrodes, respectively further attain an exalted energy density.^[^ ^3^ ^]^ However, the adaptability of SCs in flexible electronics is conceivable only if they are fabricated with high flexible electrodes. Conductive textile/fabric‐based substrates with pliable fibers and interwoven texture have been fascinated as propitious current collectors to fabricate flexible SCs because of their impressive shape resiliency, high electrical conductivity, and lightweight.^[^ ^4^ ^]^ Of several conductive textiles/fabrics, carbon fabric (CF) exhibits high mechanical strength, chemical stability, corrosion resistivity, and thermal conductivity in addition to the above stated features.^[^ ^5^ ^]^ Considering these traits of CF, directly preparing active materials on its surface by exterminating binders and additives could be the most preeminent tactic.

Apart from the storage of energy, producing green energy sources is also indispensable in the scenario of expeditious consumption of fossil fuels and environmental concerns.^[^ ^6^ ^]^ Producing hydrogen (H_2_) by electrochemical water‐splitting technology without the discharge of carbon monoxide into the environment is believed as an ideal source for future energy. The water‐splitting process consists of two core redox reactions, i.e., oxygen evolution reaction (OER) and hydrogen evolution reaction at the anodic and cathodic sides, respectively.^[^ ^7^ ^]^ Since the rate of H_2_ production relies on the OER performance of anode during the overall water‐splitting process, the competent catalyst with high electrokinetics, long durability, and low consumption of energy should have been fabricated.^[^ ^8^ ^]^ It is familiar that oxides of noble metals like iridium and ruthenium are the leading catalysts to drive high OER performance.^[^ ^9^ ^]^ Nevertheless, the constraints such as high price, detrimental effect on the environment, and compromised durability limit their wide usage as catalysts for OER.^[^ ^10^ ^]^ Therefore, the catalysts with high OER activity should have been prepared without the inclusion of noble metals by a facile synthesis route to recede the eco‐scarcity and the production cost.

Transition metal phosphides (TMPs) with high electrochemical activity, metalloid structure, high chemical stability, and multielectron orbitals have recently been captivated as potential electrode candidates in energy storage, water splitting, etc.^[^ ^11^ ^]^ Particularly, the TMPs made up of commendable transition metals of cobalt (Co) or nickel (Ni) have been explored as electrode materials in the energy storage field and potential catalysts for water‐splitting technology.^[^ ^12^ ^]^ Co and Ni elements generally exhibit high redox chemistry, multivalence states, high electrical conductivity, and good durability, and more importantly, they have closer atomic and electronic structures. As a result, CoNi‐based materials can demonstrate good electrochemical activity and more stability toward corrosion. Therefore, fabricating the binary TMPs with both Co and Ni elements in the form of a composite may endow substantial performance compared to their monometallic counterparts by exploiting the collaborative effects of respective single TMPs.^[^ ^13^ ^]^ Here, the introduction of the phosphorous element in the CoNi‐matrix enhances the number of active sites, electrical conductivity, and density of defect sites by modifying the electronic structure. Especially in the electrocatalytic process, the phosphorous element can serve as a base to absorb the protons. Moreover, the Co‐Ni phosphides show improved electrical conductivity due to their metallic properties.^[^ ^14^ ^]^ Morphology is another crucial parameter to derive the enhanced performance in SC and OER studies. The characteristics of morphological size, surface area, porosity, and their rational construction can govern the accessibility of active sites, mass diffusion, and charge‐transport process. Downsizing the size of any morphology to the nanoscale range will considerably increase the surface area, which in turn defines the number of electrochemically active sites. Here, the active sites and the electrical conductivity of active materials increase the rate of electrochemical reactions and the charge‐transfer process, respectively. Specifically, one‐dimensional (1D) structures typically exhibit high surface area so that their entire active sites can participate in electrochemical reactions. Fabricating the hybrid nanoarchitecture (HNA) would be beneficial as two kinds of morphologies are complemented each other with their respective intriguing features. On the other hand, the direct construction of active materials on the current collector promotes fast electrokinetics.

Inspiring from the above discussion, we directly synthesized the CoNiP‒CoP_2_ (CNP‐CP) materials on a highly flexible CF substrate with the 3D‐on‐1D HNA of cashew fruit‐like nanostructures and nanocones (NCs) by a one‐step hydrothermal method. It is believed that this is the first report on the prepared CNP‒CP material especially in the form of nanocashew fruit morphology by the synthesis method. The impact of growth temperature on this new morphology and the role of solitary TMPs (i.e., CoP_2_ and Ni_3_P) is unraveled comprehensively. Thanks to high redox chemistry, morphology, and fast electrokinetics, the CNP‒CP 3D‐on‐1D HNA electrode demonstrated dominant energy storage performance as well as good OER activity. Also, in situ electrochemical reactions in the CNP‒CP material of the hybrid cell were investigated by an operando X‐ray absorption near‐edge structure (XANES) analysis. Furthermore, it showed steady electrocatalytic activity over 24 h of the stability test performed at 10 mA cm^–2^. Remarkably, the CNP‒CP 3D‐on‐1D HNA‐based hybrid cell exhibited exceptional durability over 30 000 cycles. The practicability of this CNP‒CP 3D‐on‐1D HNA electrode is also disclosed by fabricating a quasi‐solid‐state hybrid cell, followed by energizing electronic components.

## 2 Results and Discussion

**Figure 1:**
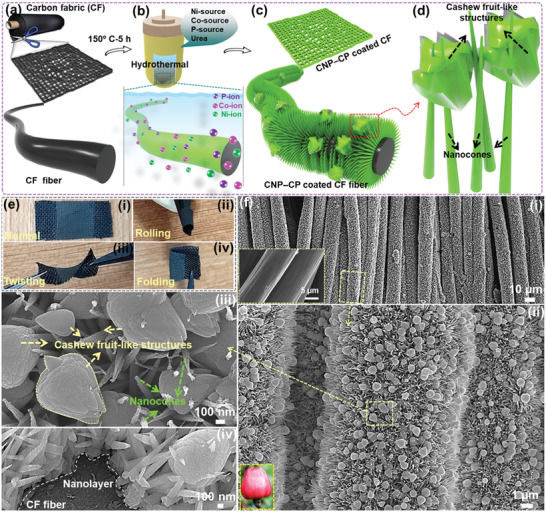
a–d) Schematic description for the straightaway deposition of the CNP‒CP‐150 material. e) Photographic images of the CNP‒CP‐150 material grown CF substrate demonstrating different shape resilience conditions. f‐i–iv) Low‐ to high‐magnification FE‐SEM images of the CNP‒CP‐150 material grown CF substrate. Inset in (f‐i) shows the FE‐SEM image of the bare CF substrate.

The preparation of straightaway grown CNP‒CP material with HNA is schematically interpreted in **Figure** **1**. First and foremost, the current collector is one of the main requirements in the construction of energy storage systems. Until now, different conducting substrates like nickel foam/foil, copper foam/foil, titanium foil, stainless steel foil, graphite sheet, etc. have been employed as current collectors.^[^ ^15^ ^]^ However, these substrates are not able to have a significant impact on the flexible energy storage systems due to their rigidness and nonflexible properties. In contrast, textile‐based substrates bestow shape resilience features, that is, they are bendable, foldable, twistable, and even rollable. The CF substrate comprising of all these features has been then elected as the current collector to design the flexible SCs in the scenario of recent advances in flexible and portable electronics. As shown in Figure 1a, the CF substrate was sliced into the required size. To prepare the CNP‒CP growth solution, the salts of Ni, Co, and P along with urea were dissolved in demineralized water (DMW) in Teflon liner. The CF substrate attached to a glass slide was then dipped in the above‐prepared growth solution, followed by heating in the autoclave. Under the reaction condition of 150 °C for 5 h in the typical hydrothermal process, the liberated metal cations (Co^2+^ and Ni^2+^) from their respective salts react with H_2_PO_2_^–^ ions and precipitate as a Co‐Ni phosphide thin film on the surface of CF fibers (Figure 1b). The reaction temperature further accelerates the precipitation of Co‐Ni phosphide nuclei due to the gradual increment of basicity in the reaction medium. In this process, the urea in the growth solution serves as a surfactant to enable the vertical growth of Co‐Ni phosphide nuclei, resulting in the formation of NCs, as displayed in Figure 1c. Interestingly, the vast number of cashew fruit‐like nanoparticles were additionally deposited on these NCs under the growth conditions, as depicted in Figure 1d. The reason is likely ascribed to the accessibility of numerous Ni^2+^, Co^2+^, and H_2_PO_2_^–^ ions even after the formation of NCs, and their inability to diffuse toward the CF fiber surface. Consequently, these excessive ions may treat the NCs as the platforms and are deposited randomly on them without particular orientation. The photographic image of the CNP‒CP material (named as CNP‐CP‐150) grown CF substrate is presented in Figure 1e‐i. Other photographic images, i.e., Figure 1e‐ii–iv shows the different resilience conditions of the active material grown substrate. Legibly, the CNP‒CP‐grown CF substrate efficiently exhibited different physical deformation conditions such as rolling, bending, and even twisting.

The prepared sample was probed by using a field‐emission scanning electron microscope (FE‐SEM) to explore the morphology of the grown CNP‒CP material. The FE‐SEM image in the inset of Figure 1f‐i revealed the smooth surface of CF fibers. In contrast, the surface of these CF fibers became rough after the hydrothermal process (Figure 1f‐i), which indicates the successful deposition of the CNP‒CP material on their surface. Besides, even distribution of the CNP‒CP material on CF fibers without any surface cracks can be perceived. Figure 1f‐ii shows the magnified FE‐SEM image. This FE‐SEM image revealed the growth of HNA composed of NCs along with nanoparticles. Further magnified FE‐SEM image in Figure 1f‐iii uncovered the precise shape of HNA. Here, the NCs with sharp top edges were vertically grown from the CF fiber, which can offer a high surface area. Moreover, other nanoparticles were arbitrarily deposited on these NCs, and their profiles look like a cashew fruit as displayed in the inset of Figure 1f‐ii. The size of these nanocashew fruits (NCFs) varies from 850 to 900 nm. Another FE‐SEM image in Figure 1f‐iv revealed the growth of NCs from CF fibers. From this image, the vertical length of an NC was observed to be ≈1.1 µm. Moreover, the width of NCs was gradually decreased from their base to the top. A layer‐like coating can be perceived on the CF fiber, which may be a seed layer of the CNP‒CP material. The NCs are subsequently grown from this layer, followed by the deposition of NCFs. The same CNP‒CP material was also prepared at distinct growth temperatures of 130 and 170 °C for the same reaction time of 5 h to scrutinize the change in the morphology concerning temperatures. The CNP‒CP materials prepared at the growth temperatures of 130, 150, and 170 °C are named CNP‒CP‐130, CNP‒CP‐150, and CNP‒CP‐170, respectively. The FE‐SEM images of CNP‒CP‐130 and CNP‒CP‐170 samples are displayed in Figures S1a and S2a (Supporting Information), respectively. As depicted in Figure S1a (Supporting Information), all the CF fibers were uniformly coated with the CNP‒CP‐130 material. From the magnified FE‐SEM image in Figure S1b (Supporting Information), only NCs that were grown vertically from the CF fibers can be seen, but no NCFs are noticed. This would likely be attributed to the inadequate growth temperature for the deposition of NCFs. When the growth temperature was increased to 170 °C, the NCs as well as the NCFs were grown on the CF fibers (Figure S2b, Supporting Information). Nevertheless, several cracks were evolved as shown in Figure S2a (Supporting Information) due to the overgrowth temperature. In addition to these samples, solitary metal phosphides such as nickel phosphide (NP‐150) and cobalt phosphide (CP‐150) materials were also synthesized at 150 °C for 5 h to explore the impact of distinct metal salts on the HNA. Figure S3 (Supporting Information) shows the FE‐SEM images of the solitary CP‐150 sample, which exhibited the randomly oriented nanowire morphology. On the other hand, nanosheet morphology was observed for the solitary NP‐150 sample (Figure S4, Supporting Information). Differently, the presence of both salts of Ni and Co in the reaction system enables the growth of both NCs and NCFs in the CNP‒CP‐150 sample.

**Figure 2:**
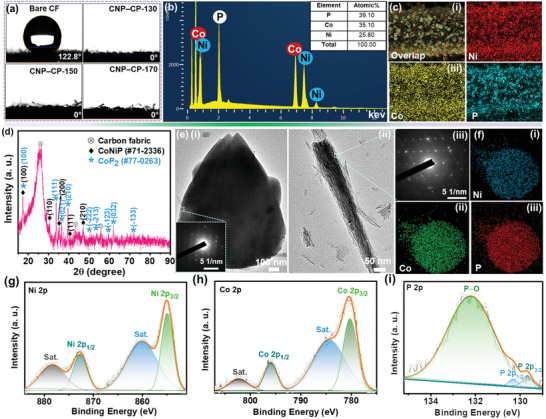
a) Contact angle measurement results of the bare CF, CNP‒CP‐130, CNP‒CP‐150, and CNP‒CP‐170 samples. b) EDS spectrum, c) elemental mapping images, and d) XRD pattern of the CNP‒CP‐150 sample. TEM images of the e‐i) NCF and ii) NC. e‐iii) SAED pattern of the NC. f) Elemental mapping images recorded from the TEM measurement. HR XPS spectra of g) Ni 2p, h) Co 2p, and i) P 2p elements. Inset in (e‐i) shows the SAED pattern of the NCF.

The contact angle measurement was performed with DMW as the solvent on the bare CF and different CNP‒CP materials prepared at the growth temperatures of 130, 150, and 170 °C. As presented in **Figure** **2**a, the bare CF substrate revealed a high contact angle of ≈123°, representing its hydrophobicity nature. Differently, all other CNP‒CP samples prepared at different growth temperatures demonstrated 0° of contact angle. The high hydrophilicity nature of CNP‒CP samples especially assists in an improvement of interaction between the active material and electrolyte while measuring their electrochemical properties. Thus, the active material becomes wet completely by the electrolyte, leading to plentiful electrochemical reactions. Energy‐dispersive X‐ray spectroscopy (EDS) analysis was performed on the CNP‒CP‐150 sample to investigate the elemental composition in the prepared active material. The resulting EDS spectrum in Figure 2b represented the elemental peaks of Co, Ni, and P. Besides, the overlay image and other mapping images (Figure 2c) endorsed the even dissemination of all the Co, Ni, P elements on the obtained morphology. X‐ray diffraction (XRD) analysis is a basic and powerful tool to unveil the phase and crystallinity of the prepared materials. Therefore, the XRD analysis was performed on the CNP‒CP‐150 sample. From the obtained diffraction spectrum plotted in Figure 2d, a broad peak with high intensity detected at ≈25.8° and the other two peaks at ≈43.4° and ≈55.7° can be noticed, which are related to the CF substrate. While other sharp diffraction peaks are the characteristic peaks of anatase CoNiP (#71‐2336) and CoP_2_ (#77‐0263) phases. All the (hkl) planes of each phase are indicated in Figure 2d at their corresponding diffraction angles. The XRD patterns of bare CP‐150 and NP‐150 samples were also recorded as presented in Figure S7 (Supporting Information). As displayed in Figure S7a (Supporting Information), the emerged diffraction peaks in the XRD pattern of bare CP‐150 confirmed the CoP_2_ (#77‐0263) phase. Meanwhile, the diffraction pattern of the bare NP‐150 sample depicted in Figure S7b (Supporting Information) recognized the Ni_3_P (#74‐1384) phase. Transmission electron microscope (TEM) analysis was employed to uncover the interior structural design of the obtained HNA. The TEM image of NCF in Figure 2e‐i showed its solid interior structure. Nevertheless, the interior part of NC looks relatively transparent (Figure 2e‐ii) when compared with the NCFs. Another TEM image of the NC is presented in Figure S6a (Supporting Information). The high‐resolution (HR) TEM image recorded at the marked area of Figure S6b (Supporting Information) reveals several crystal sites. Since the crystalline structure of the CoP_2_ phase is dominant in the material which was confirmed from the XRD pattern, other crystal sites are rarely observed/disrupted. But, the lattice planes of the CoNiP phase are also observed along with those of CoP_2_. The lattice fringes with the *d*‐spacing of ≈0.262 nm correspond to the (111) plane of the CoP_2_ phase, whereas other lattice fringes with the *d*‐spacing of ≈0.19 are ascribed to the (210) plane of the CoNiP phase. More descriptions of Figure S6 can be found in the Supporting Information. The co‐existence of both phases is also confirmed by the TEM results. Owing to both phases, heterojunctions are formed as displayed in Figure S6b (Supporting Information), which promotes faster electron transfer. Besides, the electronic structure of active sites of materials can be modulated. These aspects decrease the charge transfer resistance, followed by the enhancement in the performance of SC and electrocatalytic activity as well.^[^ ^16^ ^]^ Moreover, two phases with their respective active sites synergistically contribute to the total performance due to their corresponding features.^[^ ^17^ ^]^ The selected area electron diffraction (SAED) patterns of NCF and NC were recorded at the marked places to assess the crystallinity of the material, which are displayed in the inset of Figure 2e‐i,iii, respectively. The TEM results matched well with the XRD data, which showed sharp and intense peaks for CoP_2_ and moderate/weak peaks for CoNiP. Moreover, all the bright dots in Figure 2e‐iii are not in perfect order. Thus, the material exhibits two phases. The EDS analysis associated with the TEM chamber was further carried out to examine the elemental composition. Consistent with Figure 2b, the EDS spectrum presented in Figure S5 (Supporting Information) also exhibited the peaks regarding Co, Ni, and P elements. The even diffusion of these three elements over the HNA (Figure S5, Supporting Information and Figure 2f) further endorsed the successful formation of CNP‒CP material in the form of NCFs and NCs. The surface chemistry and valence states of the CNP‒CP‐150 material was probed by the X‐ray photoelectron spectroscopy (XPS) analysis. The recorded survey scan spectrum showed Ni 2p, Co 2p, and P 2p peaks, as displayed in Figure S8 (Supporting Information). The HR Ni 2p XPS spectrum is fitted with four peaks, in which two peaks at ≈855 and ≈872.7 eV are assigned as Ni 2p_3/2_ and Ni 2p_1/2_ energy levels (Figure 2g), respectively. The other two peaks noticed at ≈860 and ≈878.3 eV are their respective satellite peaks (indicated as Sat.). Figure 2h shows the fitted HR Co 2p XPS spectrum, which also demonstrated a spin‐orbit splitting into Co 2p_3/2_ (≈780.5 eV) and Co 2p_1/2_ (≈796 eV) energy levels with their respective Sat. peaks. The binding energy values of Ni 2p_3/2_ and Co 2p_3/2_ peaks are higher than those of the metallic Ni and Co, which signifies the existence of the oxidized Ni and Co species in the prepared material.^[^ ^18^ ^]^ The P 2p spectrum in Figure 2i exhibited a broad peak at 132.2 eV, which explains the P—O bond because of air exposure. The other two peaks embarked at 130.3 and 129.6 eV are the characteristic peaks of P 2p_1/2_ and P 2p_3/2_ states, respectively.^[^ ^13^, ^19^ ^]^ Overall, all the obtained results from the versatile physical characterization comprehensively corroborated that the prepared material is composed of mixed‐phase, i.e., CoNiP and CoP_2_.

**Figure 3:**
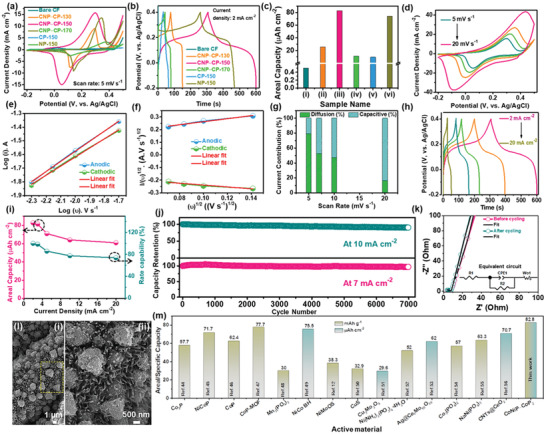
Comparison of a) CV curves at 5 mV s^–1^, b) GCD curves at 2 mA cm^–2^, and c) areal capacity values of the bare CF, CNP‒CP‐130, CNP‒CP‐150, CNP‒CP‐170, CP‐150, and NP‐150 electrodes. d) CV curves of the CNP‒CP‐150 electrode recorded at various sweep rates. e) Relationship between the logarithm of sweep rates and peak currents. f) Relationship between *v* ^1/2^ versus *i/v* ^1/2^. g) Diffusion‐ and capacitive‐controlled current contributions of the CNP‒CP‐150 electrode at measured sweep rates. h) GCD curves and i) areal capacity values of the CNP‒CP‐150 electrode at different current densities. j) Durability test results of the CNP‒CP‐150 electrode investigated at 7 and 10 mA cm^–2^. k) Fitted Nyquist plots of the CNP‒CP‐150 electrode before and after the durability test. l) FE‐SEM images of the CNP‒CP‐150 electrode recorded after the durability test. m) Comparison of areal and specific capacity values of the CNP‒CP‐150 electrode with the previous literature.

The electrochemical properties of all the synthesized samples were evaluated in a two‐molar aqueous potassium hydroxide (2 m KOH) electrolyte using a three‐terminal system. In this system, the Ag/AgCl electrode and Pt‐wire are employed as reference and counter electrodes, respectively. Since all the active materials are straightaway deposited on the CF substrate, they are directly employed as working electrodes. Initially, the cyclic voltammetry (CV) test was conducted on different electrodes, i.e., bare CF, CNP‒CP‐130, CNP‒CP‐150, CNP‒CP‐170, CP‐150, and NP‐150 in the potential window of −0.2 to 0.45 V at a fixed sweep rate of 5 mV s^–1^. As displayed in **Figure** **3**a, the CV response of the CF substrate looks to be a nearly straight line, revealing its negligible capacity contribution. Other electrodes exhibited legible peaks in anodic and cathodic sweeps which imply the Faradaic‐type signature in the prepared materials. Specifically, the CNP‒CP‐150 electrode demonstrated a leading redox response with higher anodic and cathodic currents as well as a larger CV area than all other electrodes. This dominant response may likely be due to the higher deposited active mass and HNA morphology. In detail, the NCFs endow supplementary active sites in addition to the NCs, which leads to the exalted electrochemical reactions. More importantly, the CoNiP and CoP_2_ active sites in the synthesized CNP‒CP‐150 electrode enable numerous reversible redox reactions in the measured potential range in the alkaline electrolyte. The corresponding equations are provided below^[^ ^2^, ^20^ ^]^

(1) $$\mathrm{CoNiP}+2OH^{-}⇌\mathrm{Co}P_{x}\mathrm{OH}+\mathrm{Ni}P_{1-x}\mathrm{OH}+2e^{-}$$

(2) $$\mathrm{Co}P_{x}\mathrm{OH}+OH^{-}⇌\mathrm{Co}P_{x}O+H_{2}O+e^{-}$$

From the CV response of individual metal phosphates such as CP‐150 and NP‐150, it is clear that the NP‐150 electrode showed high intense redox peaks when compared to those of the CP‐150 electrode. This is probably ascribed to the nanosheet morphology, relatively high redox features, and superior electrical conductivity. Next, the galvanostatic charge‐discharge (GCD) test was carried out on all the above electrodes at a fixed current density of 2 mA cm^–2^. From Figure 3b, it is once again proved that the bare CF substrate exposed trivial charge and discharge times. Nevertheless, it can efficiently serve as a current collector due to its good electrical conductivity in the development of flexible energy storage devices. All other electrodes showed notable charge‐discharge times because the reversible electrochemical reactions were executed, following the storage of generated charge. Especially, the CNP‒CP‐150 electrode disclosed longer charge and discharge times than all other electrodes. As explained previously, the NCFs and NCs synergistically bestow more electroactive sites to accomplish exalted reversible redox reactions. Consequently, the CNP‒CP‐150 electrode (represented with Figure 3c‐iii) demonstrated the highest areal capacity of 82.8 μAh cm^–2^ (calculated using Equation S1, Supporting Information) at 2 mA cm^–2^ compared to the other electrodes, as displayed in Figure 3c. Meanwhile, the areal capacity values of the bare CF, CNP‒CP‐130, CNP‒CP‐170, CP‐150, and NP‐150 electrodes were calculated to be 0.5, 25.5, 11.1, 9.5, and 74 μAh cm^–2^, respectively. All these electrodes are represented with (i), (ii), (iv), (v), and (vi) of Figure 3c, respectively. The specific capacity of the CNP‒CP‐150 was also calculated using Equation (S2) (Supporting Information) and the obtained value was 82.8 mAh g^–1^. Thus, the CNP‒CP‐150 electrode was considered as an optimal electrode to explore its in‐depth electrochemical properties. The CV curves of the CNP‒CP‐150 electrode recorded at various sweep rates from 5 to 20 mV s^–1^ are provided in Figure 3d. The redox peaks were well maintained in all the CV profiles, especially at a high scan rate of 20 mV s^–1^, indicating its good electrochemical reversibility. To uncover the dominant charge storage phenomenon in the CNP‒CP‐150 electrode, a relationship between the current response (*i*) of respective redox peaks in all the CV curves and the sweep rates (*ν*) is plotted in Figure 3e according to the power‐law relation, i.e., *i = aν^b^*. Here, *v* is the sweep rate, *i* is the peak current, and *a* and *b* are the variables. Usually, the *b* value varies between 0.5 and 1, in which *b* = 0.5 represents the dominant diffusion‐controlled mechanism. Differently, *b* = 1 signifies the dominant capacitive‐controlled mechanism. The *b* value is determined by the slope values of fitted anodic and cathodic peak current values. From Figure 3e, the *b* values were calculated as 0.71 and 0.66 for anodic and cathodic scans, respectively. These values indicate that the diffusion‐controlled process is dominant in the CNP‒CP‐150 electrode. To quantify the percentage of diffusion‐ and capacitive‐controlled currents in CV curves precisely, the power‐law equation can be modified as below^[^ ^21^ ^]^

(3) $$i_{p}=k_{1}v+k_{2}v^{1/2}$$

Here, *i*_p_ is the peak current, and *k*_1_ and *k*_2_ are the parameters. To estimate these parameters, a graph between *v^1/2^* and *i/v^1/2^* is plotted as shown in Figure 3f. From this graph, the *k*_1_ and *k*_2_ parameter values are determined by the slope and *y*‐axis intercepts of fitted lines, respectively. The estimated capacitive‐ and diffusion‐controlled currents at measured sweep rates are plotted in Figure 3g. At sweep rates of 5, 7, 10, and 20 mV s^–1^, the diffusion‐controlled contributions are calculated to be 79.5%, 52.4%, 47.4%, and 16.5%, respectively while the capacitive‐controlled contributions are noted to be 20.5%, 47.6%, 52.6%, and 83.5% at the same corresponding sweep rates. The reason for the dominant diffusion‐controlled process at a low scan rate is having enough reaction time for the CNP‒CP active species to accomplish abundant redox reactions. In contrast, this reaction time is gradually decreased with increasing the sweep rate, leading to the receding in the diffusion‐controlled contribution. The GCD curves plotted in Figure 3h exhibited voltage plateaus, which endorsed the battery‐type reaction mechanism. Using discharge curves, the areal/specific capacity values of the CNP‒CP‐150 electrode were calculated as 82.8, 81.5, 70.9, 64.1, and 61 μAh cm^–2^ (mAh g^–1^) at their corresponding current densities of 2, 3, 5, 10, and 20 mA cm^–2^ (Figure 3i). The attained capacity value of 82.8 μAh cm^–2^ is higher/comparable with the previously reported metal phosphide‐based materials, as interpreted in **Table** **1**. The durability of the CNP‒CP‐150 electrode was probed by executing the GCD cycles up to 7000 at the fixed low and high current densities of 7 and 10 mA cm^–2^, as illustrated in Figure 3j. At the end of the durability test, the CNP‒CP‐150 electrode conserved 96.9% and 90.3% of its starting capacity when it was performed at 7 and 10 mA cm^–2^, respectively. The resistances caused by the bulk electrolyte solution (*R*_s_) and the charge‐transfer process (*R*_ct_) are determined by the electrochemical impedance spectroscopy (EIS) technique. The Nyquist plots in Figure 3k were recorded before and after the durability test within the frequency range of 100 000 to 0.01 Hz in an open‐circuit potential. Moreover, the obtained EIS curves were fitted using the equivalent circuit provided in the inset of Figure 3k. Here, the circuit consists of R1, R2, CPE1, and W_0_ which represent electrolyte solution resistance (*R*_s_), charge‐transfer resistance (*R*_ct_), constant phase element, and Warburg resistance, respectively. The Nyquist plot measured before the stability test showed the *R*_s_ and *R*_ct_ values as 2.4 and 4.7 Ω cm^–2^, respectively. After the stability test, these values were noted as 2.65 and 5.1 Ω cm^–2^, respectively. There is no substantial rise in these two resistance values, implying good electrical conductivity and fast electrokinetics of the CNP‒CP‐150 material. The values of other parameters such as CPE1 and W_0_ are provided in Table S1 (Supporting Information). The postmortem analysis was explored by performing the FE‐SEM analysis on the CNP‒CP‐150 electrode after the durability test. From the FE‐SEM image in Figure 3l‐i, the dense coating of CNP‒CP‐150 material on the CF fibers can be still perceived, like in Figure 1f‐ii without any cracks, which signifies its rigid adherence on the CF fibers. Furthermore, Figure 3l‐ii verified the high structural stability of the CNP‒CP‐150 material by preserving its HNA. However, the exterior surface of NCs and NCFs was converted into hierarchical nanosheets due to the execution of unceasing redox reactions with an alkaline electrolyte. The chart in Figure 3m briefly compares the areal capacity performance of the CNP‒CP‐150 electrode with that of the previously published metal phosphides‐based materials. Table 1 describes the comprehensive areal/specific capacity comparison with previous reports. The complete electrochemical properties of other electrodes such as CNP‒CP‐130, CNP‒CP‐170, CP‐150, and NP‐150 are provided in the Supporting Information.

**Table 1:** Areal capacity performance of the CNP‒CP‐150 electrode in comparison with the previously published metal phosphide/hydroxide/oxide‐based materials

Active material	Current collector	Preparation method	Electrolyte	Test condition	Specific/areal capacity	Ref.
Co_2_P	Nickel foam	Binder‐addition	6 m KOH	1 A g^–1^	57.7 mAh g^–1^	^[^ ^29^ ^]^
NiCoP	Nickel foam	Binder‐addition	2 m KOH	4 A g^–1^	71.7 mAh g^–1^	^[^ ^30^ ^]^
CoP	Nickel foam	Binder‐addition	6 m KOH	1 A g^–1^	62.4 mAh g^–1^	^[^ ^31^ ^]^
CoP‐MOF	Nickel foam	Binder‐addition	6 m KOH	1 A g^–1^	77.7 mAh g^–1^	^[^ ^32^ ^]^
Mn_3_(PO_4_)_2_	Nickel foam	Binder‐addition	6 m KOH	0.5 A g^–1^	30 mAh g^–1^	^[^ ^33^ ^]^
NiCo BH	Nickel mesh	Binder‐free	2 m KOH	1 mA cm^−2^	75.5 μAh cm^–2^	^[^ ^34^ ^]^
Ni_2_Mo_6_O_2_S_6_	Nickel foam	Binder‐addition	2 m KOH	1 A g^–1^	38.3 mAh g^–1^	^[^ ^3d^ ^]^
CuS	Nickel foam	Biner‐addition	2 m KOH	0.5 A g^–1^	32.9 mAh g^–1^	^[^ ^35^ ^]^
Cu_3_Mo_2_O_9_	Nickel foam	Binder‐addition	1 m KOH	1 mA cm^–2^	29.6 μAh cm^–2^	^[^ ^36^ ^]^
Ni(NH_4_)_2_(PO_3_)_4_·4H_2_O	Nickel foam	Binder‐addition	6 m KOH	0.5 A g^–1^	52 mAh g^–1^	^[^ ^37^ ^]^
Ag@Ce_6_Mo_10_O_39_	Nickel foam	Binder‐addition	1 m KOH	2 mA cm^–2^	62 μAh cm^–2^	^[^ ^38^ ^]^
Co_3_(PO_4_)_2_	Nickel foam	Binder‐addition	1 m KOH	0.5 A g^–1^	57 mAh g^–1^	^[^ ^39^ ^]^
NaNi_4_(PO_4_)_3_	Graphene foam	Binder‐addition	2 m NaNo_3_	1 A g^–1^	63.3 mAh g^–1^	^[^ ^40^ ^]^
CNTs@CeO_2_ HTs	Nickel foam	Binder‐addition	1 m KOH	1 mA cm^–2^	70.7 μAh cm^–2^	^[^ ^41^ ^]^
**CoNiP‐CoP_2_**	**Carbon fabric**	**Binder‐free**	**2 m KOH**	**2 mA cm^–2^**	**82.8 μAh cm^–2^ & 82.8 mAh g^–1^**	**This work**

**Figure 4:**
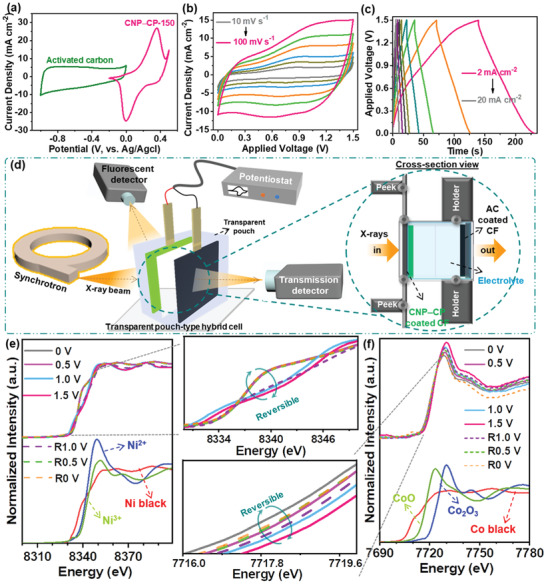
a) CV curves of the CNP‒CP‐150 and AC electrodes measured at their corresponding voltage windows to estimate the optimum voltage window of a hybrid cell. b) CV and c) GCD curves of the hybrid cell within the applied voltage window of 0–1.5 V. d) Schematic illustration for the *Operando* XANES measurement of the hybrid cell. e) Ni K‐edge and f) Co K‐edge XANES spectra of the hybrid cell.

The achieved good capacity and long durability characteristics of the CNP‒CP‐150 electrode were further explored in a two‐terminal system by assembling a hybrid cell. The scheme in Figure S14a (Supporting Information) exemplifies the assembling of the hybrid cell, in which the CNP‒CP‐150, microporous filter paper, activated carbon loaded CF substrate (AC@CF), and 2 m KOH solution are served as the positive electrode, separator, negative electrode, and electrolyte, respectively. Before the assembly of the hybrid cell, balancing the masses of both electrode materials is obligatory to achieve high and stable performance. Accordingly, the optimal mass of the AC material was derived from Equation (S7) (Supporting Information), which is ≈1.5 mg cm^–2^. Next, the AC@CF electrode was fabricated with the procedure described in the Supporting Information. After assembling the hybrid cell, determining its operational voltage regime is another decisive parameter to inhibit the overcharging, which may cause the gradual deterioration of the active material over a long number of cycles. The comparative CV curves of the CNP‒CP‐150 and AC@CF electrodes recorded at a fixed sweep rate of 10 mV s^–1^ within their particular potential windows are shown in **Figure** **4**a. From this figure, the operational voltage regime of the hybrid cell can be predicted to be 0–1.5 V. To certify this statement, the CV (at 40 mV s^–1^) and GCD (at 5 mA cm^–2^) tests were performed on the hybrid cell under constant test conditions by gradually extending its voltage regime from 0–0.8 to 0–1.5 V, as depicted in Figure S14b,c (Supporting Information), respectively. No substantial deviations were noticed from these two plots, which confirmed that the operational voltage regime of the hybrid cell was 0–1.5 V. The same CV and GCD tests of the hybrid cell were performed at various test conditions within its optimized voltage regime (0–1.5 V). From the corresponding data plotted in Figure 4b,c, both CV and GCD profiles well maintained their outlines even at high test conditions, revealing well‐balancing of charges and excellent reversibility of the hybrid cell.

To gain an insight into the correlation between the enhanced capacitance of CNP‐CP and change in its electronic structure, X‐ray absorption near‐edge structure (denoted as XANES) measurements on the Ni and Co K‐edge were performed under in situ/operando conditions. The scheme in Figure 4d illustrates the electrochemical set‐up with integrated in situ/operando XANES analysis. The magnified image in Figure 4d represents the cross‐sectional view of the hybrid cell while enacting the XANES analysis. The XANES analysis was carried out on the hybrid cell when measuring the CV analysis by applying a low sweep rate of 5 mV s^‒1^ at the applied cell voltages of 0, 0.5, 1.0, and 1.5 V in the forward sweep. Meanwhile, in the backward sweep, the same analysis was carried out in the reverse order of voltage points, i.e., 1.0, 0.5, and 0 V (named as R1.0 V, R0.5 V, and R0 V, respectively). The Ni K‐edge XANES spectra of the hybrid cell in Figure 4e exhibited a white line shape similar to the Ni black as a reference, which indicates that the metallic Ni remains dominant even in the real‐time electrochemical measurement. On the other hand, it is confirmed that the pre‐edge region slightly shifts in the high energy direction as the applied cell voltage increases from 0 to 1.5 V. This shift implies the increment in the valency of Ni element due to the oxidation process with alkaline electrolyte ions.^[^ ^22^ ^]^ When the sweep rate is reversed, the energy of Ni K‐edge XANES spectral lines obtained at R1.0 V, R0.5 V, and R0 V is gradually decreased, indicating the reduction in the Ni valency. Moreover, the overlapping of Ni K‐edge XANES spectral lines obtained at 0 V and R0 V indicates good electrochemical reversibility. The Co K‐edge XANES spectra displayed in Figure 4f also revealed the shifting in the peak of the white line toward higher energy when the applied voltage increased from 0 to 1.5 V, endorsing the gradual rise for the oxidation state of Co.^[^ ^23^ ^]^ However, the tendency of the Co K‐edge peak position shifting toward lower energies could be noticed when the XANES measurement was performed at the R1.0 V, R0.5 V, and R0 V sequentially, which interprets the reduction process of Co element in the backward sweep. Interestingly, the oxidation state of Co showed more than 3+ state from the initial OCV, whereas Ni maintained the metallic phase. On the whole, the in situ/operando XANES measurement validated the reversible changes in the valency of Co and Ni elements in the CNP‒CP material during the real‐time electrochemical measurement.

**Figure 5:**
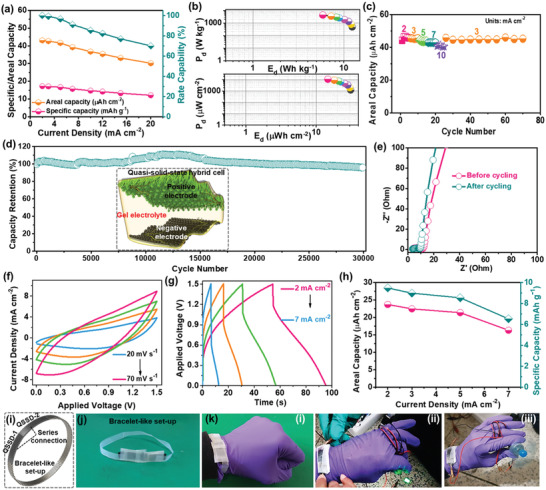
a) Specific/areal capacity values at different current densities, b) specific/areal energy and power density values of the hybrid cell. c) Rate performance test and d) durability test results of the hybrid cell. e) Nyquist plots of the hybrid cell obtained before and after the durability test. f) CV curves at various sweep rates, g) GCD curves, and h) areal/specific capacity values of the QSSD. i–k) Bracelet‐like set‐up designed to demonstrate the practicability of the QSSD. Powering of k‐ii) an LED and iii) a motor fan with QSSDs arranged in a bracelet‐like set‐up. Inset in (d) shows the fabrication of QSSD.

The areal and specific capacitances of the hybrid cell were estimated at various current densities using Equations (S1) and (S2) (Supporting Information), respectively. At 2 mA cm^–2^, the hybrid cell delivered its maximum areal capacity of 43 μAh cm^–2^ (17.23 mAh g^–1^), and it still showed 30.1 μAh cm^–2^ (12 mAh g^–1^) at a high current density of 20 mA cm^–2^ with a good rate capability of 78.3% (**Figure** **5**a). Other vital characteristics of the hybrid cell such as areal energy density (*E*_d_) and power density (*P*_d_) were calculated using Equations (S4) and (S6) (Supporting Information), respectively. By exploiting the high redox chemistry of the CNP‒CP material and novel HNA, the hybrid cell demonstrated its maximum *E*_d_ and *P*_d_ values of 31 μWh cm^–2^ and 10.9 mW cm^–2^, respectively, as presented in Figure 5b. The maximum specific energy (Equation S5, Supporting Information) and power density values were also evaluated as 12.4 Wh kg^–1^ and 4354 W kg^–1^, respectively, which are also plotted in Figure 5b. The comparative energy density values of our hybrid cell were compared with reported literature as shown in Table S2 (Supporting Information). The rate performance test of the hybrid cell was also conducted by measuring five GCD cycles at the current densities of 2, 3, 5, 7, and 10 mA cm^–2^. The specific capacitances were evaluated from all the discharge curves and plotted in Figure 5c. These values decreased gradually when the current density increased. When the current density returned to 3 mA cm^–2^, the hybrid cell potentially recovered almost the same capacitance values that were obtained at the same test condition initially. Moreover, the hybrid cell retained nearly the same value for 50 more cycles, unveiling its high rate performance. The durability test was then implemented at a fixed current density of 7 mA cm^–2^. Figure 5d reveals the extraordinary durability of the hybrid cell. It is worth mentioning here that the hybrid cell demonstrated 95.8% of retention even after executing 30 000 unending charge‐discharge cycles. The *R*_s_ and *R*_ct_ values of the hybrid cell were examined before and after the durability test using the EIS technique. The resultant Nyquist plots are depicted in Figure 5e. After the durability test, a slight reduction in *R*_s_ and *R*_ct_ values is observed, which is likely due to the activation of the HNA. After the durability test, FE‐SEM, XRD, and XPS analyses were performed on the CNP‒CP‐150 electrode (of the hybrid cell), and the obtained results are presented in Figures S14d and S15 (Supporting Information). The FE‐SEM image in Figure S14d (Supporting Information) further endorsed the robust adherence of the morphology to the current collector. As shown in Figure S15a (Supporting Information), the XRD spectrum still showed almost all the peaks related to CoNiP and CoP_2_ phases, which indicates good stability of the material. However, the intensity of all these peaks is relatively diminished. This specifies the decreased crystallinity of the material as the rough surface of the HNA converted into nanosheets. The FE‐SEM image in Figure S14d‐ii (Supporting Information) shows several thin nanosheets on the hybrid nanostructures. Typically, the metal phosphides would convert into metal (oxy)hydroxides at the surface of respective morphology during the long‐term cycling process in an alkaline electrolyte. Since the XRD analysis is not surface‐sensitive to discern these changes, the XPS analysis was carried out on the same sample. The XPS survey scan spectrum in Figure S15b (Supporting Information) shows Ni 2p, Co 2p, and O 1s related peaks. However, the peak related to the P 2p disappeared due to its dissolution in akaline electrolyte and suggest the formation of metal (oxy)hydroxides. The Co 2p spectrum in Figure S15c (Supporting Information) shows the peaks of Co 2p_3/2_ and Co 2p_1/2_ at 780.1 and 795.3 eV, respectively. But, the intensity of satellite peaks is substantially decreased when compared with the same Co 2p spectrum of fresh sample, representing the formation of Co(III) with low‐spin.^[^ ^24^ ^]^ In the Ni 2p spectrum (Figure S15d, Supporting Information), the spin energy separation between the Ni 2p_3/2_ and Ni 2p_1/2_ energy levels is noticed as ≈17.62 eV, which manifests the formation of the NiOOH phase at the surface. But, no apparent signal in the P 2p spectrum (Figure S15e, Supporting Information) further validifies the surface oxidation of the active material.^[^ ^25^ ^]^ The O 1s peak in the survey spectrum with a slight high intensity further confirms this tendency. The O 1s HR XPS spectrum in Figure S15f (Supporting Information) is deconvoluted into three peaks at ≈532.8, ≈531.1, and ≈529.3 eV which signify defect sites, hydroxyl species, and metal‐oxygen species, respectively.^[^ ^24^ ^]^ Thus, the XRD and XPS analyses reveal the in situ formation of the (Ni,Co)OOH phase during the stability test.

Considering the impressive energy storage properties of the hybrid cell, a quasi‐solid‐state device (QSSD) was also fabricated to prevent liquid‐electrolyte leakage from the cell. The arrangement of electrodes in a QSSD is similar to that of an aqueous hybrid cell. But, the aqueous electrolyte and the separator were replaced by poly(vinyl alcohol) (PVA) and KOH gel‐electrolyte. This PVA‐KOH gel layer serves as both electrolyte and separator. Inset in Figure 5d shows the schematic illustration for the fabrication of a QSSD. The CV (Figure 5f) and GCD (Figure 5g) profiles of the QSSD recorded at various test conditions exhibited good charge‐storage properties and electrochemical reversibility. At 2 mA cm^–2^, the QSSD delivered its maximum specific capacity of 23.7 μAh cm^–2^ (9.5 mAh g^–1^). This device even retained 16.3 μAh cm^–2^ (6.5 mAh g^–1^) at a high current density of 7 mA cm^–2^, revealing its noteworthy rate capability of 80.1% (Figure 5h). The practicability of the fabricated QSSD was also explored by designing a bracelet‐like set‐up. As shown in Figure 5i, the bracelet‐like set‐up was designed by combining two QSSDs in a series connection to extend the voltage range, followed by wrapping with scotch tape. The photographic images of the bracelet‐like set‐up are portrayed in Figure 5j,k‐i. After charging, the QSSD set‐up potentially powered a green light‐emitting diode (LED) and even a motor fan as depicted in Figure 5k‐ii–iii, respectively.

**Figure 6:**
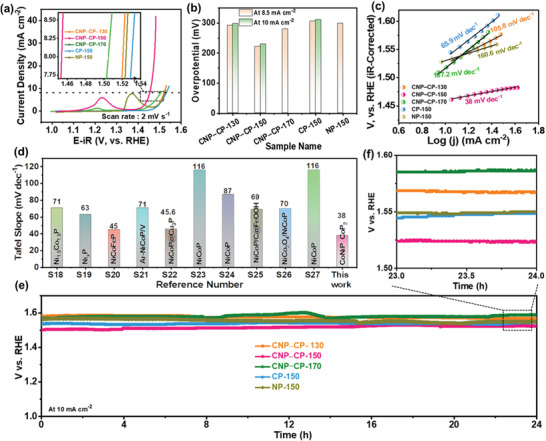
OER activity of the prepared catalysts. a) LSV profiles of the CNP‒CP‐130, CNP‒CP‐150, CNP‒CP‐170, CP‐150, and NP‐150 catalysts obtained at a low sweep rate of 2 mV s^–1^. b) OP values of different catalysts that are required to drive the current density of 8.5 and/or 10 mA cm^–2^. c) Tafel slopes of all the prepared catalysts. d) Comparison of Tafel slope values of the CNP‒CP‐150 catalyst with the previous reports. e,f) Chronopotentiometry test performed on the CNP‒CP‐150 catalyst at 10 mA cm^–2^ for 24 h.

The electrocatalytic activity of the CNP‒CP‐150 sample was also investigated for the OER in alkaline media and compared with the other prepared catalysts. This investigation was carried out in a conventional three‐terminal set‐up as mentioned above. The potentials in which the electrocatalytic activity is measured were calibrated according to the reference reversible hydrogen electrode (RHE). The linear sweep voltammograms (LSVs) of all the prepared catalysts recorded at a slow sweep rate of 2 mV s^–1^ are depicted in **Figure** **6**a. It is legible that the CNP‒CP‐150 catalyst unveiled its highest OER activity at a low onset overpotential (OP) value compared to all the other catalysts. The mass activity was also investigated by normalizing all the LSV profiles of the catalysts with their respective mass loadings. As shown in Figure S16 (Supporting Information), the CNP‒CP‐150 catalyst still exhibited superior performance to all other catalysts. The OP values of all the catalysts are depicted as a bar diagram in Figure 6b. The CNP‒CP‐150 catalyst requires only an OP value of 222 mV to deliver the geometric current density of 8.5 mA cm^–2^. Meanwhile, the other catalysts such as CNP‒CP‐130, CNP‒CP‐170, CP‐150, and NP‐150 showed relatively higher OP values of 292, 280, 306, and 299 mV to deliver the same current density. Since some of the catalysts were unable to deliver 10 mA cm^–2^, the onset OP values of all the catalysts were considered at the current density of 8.5 mA cm^–2^. Nevertheless, the CNP‒CP‐150 catalyst delivered the current density of 10 mA cm^–2^ at a lower OP value of 230 mV. Small anodic humps in the LSVs of some catalysts could be noticed in the regions of ≈1.15–1.3 V and ≈1.3–1.45 V versus RHE, which is ascribed to the oxidation of Ni and/or Co species (Ni^2+^/Ni^3+^ and Co^2+^/Co^3+^). However, the peak center of the CNP‒CP‐150 catalyst was observed at a lower potential, i.e., ≈1.23 V versus RHE than that of the NP‐150 catalyst (≈1.37 V vs RHE), specifying the alteration of NP electronic structure by the existence of Co species.^[^ ^26^ ^]^ The Tafel slopes of all the catalysts were derived from the Tafel equation (*η* = *b* log *j* + *a*, where *j* is the current density and *b* is the Tafel slope) to explore the kinetics of the catalysis reaction. From Figure 6c, a minimum Tafel slope of 38 mV dec^–1^ was perceived for the CNP‒CP‐150 catalyst, which is inferior to the Tafel slopes of the CNP‒CP‐130 (105.8 mV dec^–1^), CNP‒CP‐170 (167.2 mV dec^–1^), CP‐150 (65.9 mV dec^–1^), and NP‐150 (160.6 mV dec^–1^) catalysts. This inferior slope value reveals a superior electrokinetic of the CNP‒CP‐150 catalyst while performing the OER activity. Moreover, the obtained Tafel slope of the CNP‒CP‐150 catalyst is lower/comparable than those of previously fabricated various catalysts, as shown in Figure 6d and Table S3 (Supporting Information). The structural merits of the 3D‐on‐1D HNA in achieving of the impressive performance in both SC and OER applications are described as follows: the vertically emerged NCs offer a high surface area, which exalts the accessibility of active sites to electrolyte ions. The free‐space among them facilitates the deep diffusion of electrolyte ions, which leads to the activation of more active sites. Consequently, the number of electrochemical reactions will be increased, followed by the enhancement of SC/OER performance. Moreover, these NCs also serve as electron superhighways to transfer the generated electrons to an external load. On the other hand, the NCFs anchored over these NCs further improve active sites, that enhances the electrochemical reaction rate. The NCs and NCFs are, therefore, well complement each other in the enhancement of overall performance. Additionally, the structural sustainability of any morphology in the SC/OER stability test is highly desirable. As presented in Figure 3l and Figure S14d (Supporting Information), these two morphologies well retained their outlines without collapsing even after the long‐term stability test. The chronopotentiometry experiment was then performed on all the catalysts by applying a steady current density of 10 mA cm^–2^ to evaluate the electrocatalytic stability. On 24 h of stability test, the CNP‒CP‐150 catalyst remained active without substantial change (Figure 6e). The reason for the slight increment in the voltage value of the CNP‒CP‐150 catalyst could be the gradual formation/accumulation of gas bubbles on the catalyst active area, which might hinder the catalytic activity.^[^ ^27^ ^]^ As disclosed in Figure 6f, the potential value of the CNP‒CP‐150 catalyst is still lower than those of all the other catalysts even after 24 h, indicating its superior electrocatalytic activity. After the stability test, XPS analysis was carried out on the CNP‒CP‐150 catalyst to explore the changes in its phase and surface oxidation. Metal phosphides are usually converted into metal (oxy)hydroxides in an alkaline electrolyte during the OER process.^[^ ^28^ ^]^ The obtained XPS results are plotted in Figure S17 (Supporting Information). Since the OER measurement was also carried out in the same alkaline electrolyte as in the supercapacitor study, the XPS survey scan spectrum and other HR XPS spectra (Co 2p, Ni 2p, P 2p, and O 1s) of the CNP‒CP‐150 catalyst also exhibited their characteristic peaks at nearly the same binding energy values of those observed in the XPS data of the CNP‒CP‐150 electrode (Figure S15b‐e). This XPS analysis, therefore, reveals the in situ formation of the (Ni,Co)OOH phase during the OER measurement and further indicates that the “real‐active sites” of the metal phosphides‐based materials are (Ni,Co)OOH species.

**Figure 7:**
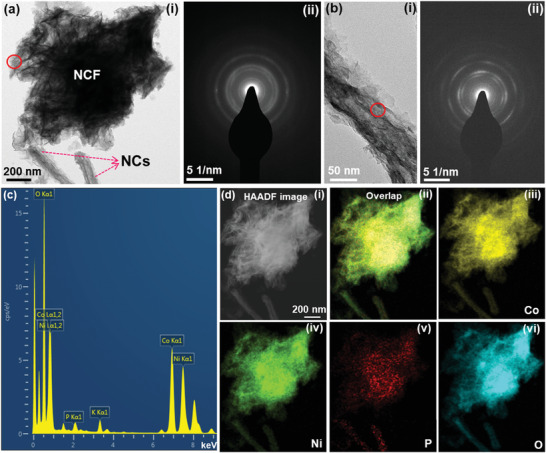
TEM results of the CNP‒CP‐150 catalyst after the stability test in the OER study. a‐i) TEM image and ii) SAED pattern of the NCF. b‐i) TEM image and ii) SAED pattern of the NC. c) EDS spectrum and d) elemental mapping images of the HNA.

The TEM analysis was further carried out on the CNP‒CP‐150 catalyst after the stability test in the OER study. As shown in **Figure** **7**a‐i, the surface of the NCF structure became transparent by transforming into thin nanosheets due to the ceaseless electrochemical reactions with an alkaline electrolyte. These nanosheets represent the formation of metal (oxy)hydroxide phase. The semi‐transparent TEM image of NC in Figure 7b also displayed the formation of several nanosheets on its surface. The SAED patterns in Figure 7a‐ii,b‐ii of NCF and NC recorded at the circled places showed the bright ring‐like patterns that signify the polycrystalline nature of newly formed nanosheets. The EDS spectrum in Figure 7c exhibited Co, Ni, P, O, and K (potassium) peaks. Here, the K peak arises due to the interaction of the material with an alkaline electrolyte. The elemental mapping images in Figure 7d revealed the uniform distribution of Co, Ni, and O elements over the morphology. Differently, the P element was barely observed on the newly formed nanosheets on the surface of morphology, which might be stripped by the alkaline electrolyte. This trend consents to the P 2p spectrum in the XPS analysis. Meanwhile, the oxygen content is observed in the same location, which also implied the metal (oxy)hydroxide phase. Overall, all the TEM results supported the XPS results and further verified the in situ formation of metal (oxy)hydroxides on the surface of the catalyst.

## 3 Conclusions

The dual metal phosphides, i.e., CoNiP‒CoP_2_, were straightaway deposited with a 3D‐on‐2D HNA on the CF substrate via a facile one‐pot hydrothermal technique. In the attained HNA, the NCs were vertically grown from the CF fibers, which themselves acted as scaffolds to grow the captivating NCFs. The temperature impact on this novel morphology was uncovered by preparing different CNP‒CP samples at the other temperatures of 130 and 170 °C. The influence of solitary metal phosphides (CP and NP) on the electrochemical performance was also explored by synthesizing them at the optimized growth temperature of 150 °C. Among all the electrodes, the CNP‒CP‐150 electrode revealed its superior electrochemical response in the energy storage as well as OER owing to the boosted redox chemistry and advantageous novel morphology. The CNP‒CP‐150 electrode delivered its highest areal capacity of 82.8 μAh cm^–2^ at 2 mA cm^–2^ and long durability until 7000 cycles. The hybrid cell with the CNP‒CP‐150 as a positive electrode also demonstrated its maximum *E*_d_ and *P*_d_ values of 31 μWh cm^–2^ (12.4 Wh kg^–1^) and 10.9 mW cm^–2^ (4354.6 W kg^–1^), respectively. From in situ/operando XANES analysis, the reversible changes in the valency of Co and Ni elements in the CNP‒CP material of the hybrid cell were validated during the real‐time electrochemical reactions. Additionally, it demonstrated outstanding durability until 30 000 cycles. On the other hand, the CNP‒CP‐150 electrode displayed a superior electrocatalytic activity by requiring a lower OP value of 222 mV among all the other electrodes to accomplish a current density of 8.5 mA cm^–2^ (230 mV for 10 mA cm^–2^). Moreover, the CNP‒CP‐150 catalyst exhibited the smallest Tafel slope and well‐accomplished an OER activity for 24 h. These results may shed a light on the derivation of mixed metal phosphides with captivating and benefitted nanoarchitecture using a one‐pot hydrothermal technique for multifunctional applications.

## 4 Experimental Section

The details of chemicals and other materials used to prepare the samples are described in the Supporting Information.

### Synthesis of Metal Phosphide Samples

The CNP‒CP HNA (i.e., NCs and NCFs) was prepared by a one‐pot hydrothermal technique. Initially, the chloride salts of cobalt (CoCl_2_·6H_2_O, 0.43 g) and nickel (NiCl_2_·6H_2_O, 0.48 g) were mixed in a Teflon liner containing 60 mL of DMW. Then, sodium hypophosphite (NaPO_2_H_2_, 0.14 g) and urea (CH_4_N_2_O, 0.47 g) were added to the above solution and this growth solution was left for about 10 min for the complete dissolution of all the chemicals. Meanwhile, the CF substrate was attached to a glass slide with the aid of Teflon tape by allowing only its 1 × 1 cm^2^ surface area to grow the active material. This CF substrate was initially oxidized in the muffle furnace to make it hydrophilic. The CF immersed growth solution was then heated in the autoclave reaction system at 150 °C and held at this temperature for 5 h. After cooling the autoclave, the CNP‒CP loaded CF substrate was cleaned with DMW several times, followed by drying in an oven overnight. Furthermore, the same CNP‒CP materials were synthesized at different growth temperatures of 130 and 170 °C for the same growth time of 5 h in an attempt to explore the impact of growth temperature. The CNP‒CP materials prepared at the growth temperatures of 130, 150, and 170 °C for 5 h were designated as CNP‒CP‐130, CNP‒CP‐150, and CNP‒CP‐170, respectively. Other samples such as CP and NP were also prepared at the same growth conditions (150 °C for 5 h) to reveal the effect of solitary metal phosphides. These two samples were named CP‐150 and NP‐150, respectively. The masses of CNP‒CP‐130, CNP‒CP‐150, CNP‒CP‐170, CP‐150, and NP‐150 samples were measured as ≈0.91, ≈1.0, ≈1.2, ≈0.5, and ≈0.9 mg cm^–2^, respectively.

The information concerning the physical characterization techniques that were employed to analyze the prepared samples is provided in the Supporting Information.

### Physicochemical Characterization

FE‐SEM (MERLIN (Carl Zeiss)) attached with EDS (MERLIN (Carl Zeiss)) apparatus was employed to check the surface morphology and elements distributed in prepared samples, respectively. A TEM (JEM‐2100F (JEOL)) instrument was used to study the internal structural properties of the samples. Crystallinity and phase purity characteristics of the samples were studied by XRD (D8 Advance (Bruker)). XPS (K‐alpha (Thermo Electron)) analysis was used to explore the valence states of elements in the synthesized samples. Contact angle measurement was performed to synthesized samples by the contact angle analyzer (SEO–Phoenix 300) to investigate their hydrophilicity nature.

### Fabrication of a Quasi‐Solid‐State Device

Initially, the PVA‐KOH gel‐polymer‐electrolyte was prepared prior to the fabrication of a QSSD. At first, ≈2 g of PVA chemical was added in 20 mL of DMW and the solution was vigorously stirred with an aid of magnetic bead for about 5 min. This solution was then heated at 80 °C until the PVA chemical dissolved in DMW completely. Subsequently, the KOH solution (2 m in 10 mL DMW) was dropwise added to the above solution and the entire solution was heated again under stirring. After some time, the gel was formed due to the evaporation of excessive water. After cooling, the gel was applied on both positive and negative electrodes. These two electrodes were then stacked one over the other and packed with a Paraffin film carefully.

## Conflict of Interest

The authors declare no conflict of interest.

## Supporting information

Supporting Information

## Data Availability

The data that support the findings of this study are available from the corresponding author upon reasonable request.
