# Supplementary material for: Unraveling CoNiP‒CoP2 3D‐on‐1D Hybrid Nanoarchitecture for Long‐Lasting Electrochemical Hybrid Cells and Oxygen Evolution Reaction
Source: Adv Sci (Weinh). 2022 Jan 22;9(8):2104877. doi: 10.1002/advs.202104877 (PMC8922135; doi:10.1002/advs.202104877)
Supplement: Supplementary file 1 — Supporting Information [file ADVS-9-2104877-s001.pdf]

ADVANCED SCIENCE
Open Access

Supporting Information

for *Adv. Sci.*, DOI 10.1002/advs.202104877

Unraveling CoNiP–CoP_2 3D-on-1D Hybrid Nanoarchitecture for Long-Lasting Electrochemical Hybrid Cells and Oxygen Evolution Reaction

S. Chandra Sekhar, Bhimanaboina Ramulu, Man Ho Han, Shaik Junied Arbaz, Manchi Nagaraju, Hyung-Suk Oh and Jae Su Yu**

ADVANCED SCIENCE
Open Access

Supporting Information

for *Adv. Sci.,* DOI: 10.1002/advs.202104877

Unraveling CoNiP–CoP_2 3D-on-1D hybrid nanoarchitecture for long-lasting electrochemical hybrid cells and oxygen evolution reaction

S. Chandra Sekhar, Bhimanaboina Ramulu, Man Ho Han, Shaik Junied Arbaz, Manchi Nagaraju, Hyung-Suk Oh, and Jae Su Yu**

Supporting Information

Unraveling CoNiP–CoP_2 3D-on-1D hybrid nanoarchitecture for long-lasting electrochemical hybrid cells and oxygen evolution reaction

S. Chandra Sekhar[a], *Bhimanaboina Ramulu*[a], *Man Ho Han*[b], *Shaik Junied Arbaz*[a], *Manchi Nagaraju*[a], *Hyung-Suk Oh*[b,c,]*, *and Jae Su Yu*[a,c,]*

[a]Institute for Wearable Convergence Electronics, Kyung Hee University, 1732 Deogyeong-daero, Gihung-gu, Yongin-si, Gyeonggi-do 17104, Republic of Korea

[b]Clean Energy Research Center, Korea Institute of Science and Technology (KIST), Hwarang-ro 14-gil 5, Seongbuk-gu, Seoul, 02792, Republic of Korea

[c]KHU-KIST Department of Conversing Science and Technology, Kyung Hee University, Seoul, 02447, Republic of Korea

*Address correspondence to hyung-suk.oh@kist.re.kr (H. -S. Oh) and jsyu@khu.ac.kr (J. S. Yu)

Section-I

1.1. Chemicals and materials

Cobalt chloride hexahydrate ($CoCl_2 \cdot 6H_2O$), sodium hypophosphite ($NaPO_2H_2$), and nickel chloride hexahydrate ($NiCl_2 \cdot 6H_2O$) were bought from Sigma Aldrich Co., South Korea. Potassium hydroxide (KOH) and urea (CH_4N_2O) were purchased from DaeJung chemicals Co., South Korea. Super P carbon black, polyvinylidene difluoride (PVDF, $-(C_2H_2F_2)_n-$), and N-methyl-2-pyrrolidone (NMP, C_5H_9NO) were received bought from MTI Korea Co., South Korea. Demineralized water (DMW) was produced from the Milli-Q water purification system in our lab. All the received chemicals were of analytical grade and used as those received. Carbon fabric substrate was purchased from CeTech Co., South Korea.

1.2. Equations

The electrochemical performance of the prepared electrodes was estimated in terms of areal capacity, areal capacitance, and areal energy/power densities in three- and two-electrode systems using the following formulae:S[1]

$$C_A = \frac{2I \int v(t)\, dt}{a \times \Delta V} \quad \text{(S1)}$$

$$C_S = \frac{2I \int v(t)\, dt}{m \times \Delta V} \quad \text{(S2)}$$

$$C_{AC} = \frac{I \times \Delta t}{a \times \Delta V} \quad \text{(S3)}$$

$$E_A = \frac{I \times \int v(t)\, dt}{a} \quad \text{(S4)}$$

$$E_S = \frac{I \times \int v(t)\, dt}{m} \quad \text{(S5)}$$

$$P = \frac{E}{\Delta t} \quad \text{(S6)}$$

where 'C_A' is the areal capacity (Ah cm^{-2}), 'C_{AC}' is the areal capacitance (F cm^{-2}), 'C_S' is the specific capacity (Ah g^{-1}), 'ΔV' is the potential window (V) after excluding potential drop, '$\int v(t)\, dt$' is an integral area, 'E_A' and 'E_S' are areal and specific energy densities (Wh cm^{-2} or Wh kg^{-1}), and 'P' is the power densities (W cm^{-2} or W kg^{-1}), respectively. 'Δt' is the discharge time (s).

The mass-balancing equation to estimate the correct mass of AC material (negative electrode) for the fabrication of the hybrid cell is given below.S[2]

$$m_- = \frac{Q_+ \times m_+}{C_- \times \Delta V_-} \quad \text{(S7)}$$

Here, m_-, C_-, and ΔV_- are the mass (g), areal capacitance (F cm^{-2}), and potential window (V) of the negative electrode, respectively, while m_+, Q_+, and ΔV_+ are the mass (g), areal capacity (Ah cm^{-2}), and potential window (V) of the positive electrode, respectively.

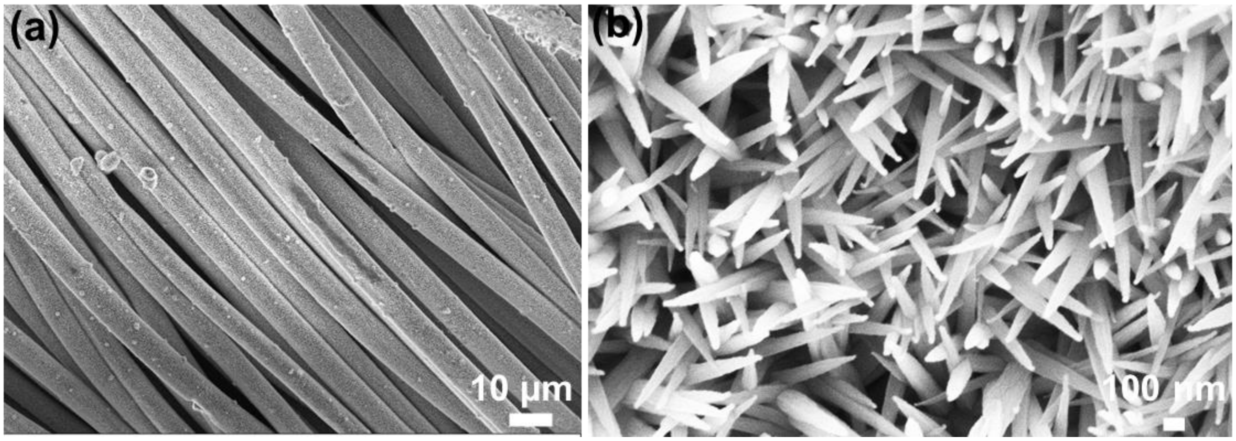

Figure S1. FE-SEM images of the CNP–CP-130 sample at (a) low and (b) high magnifications.

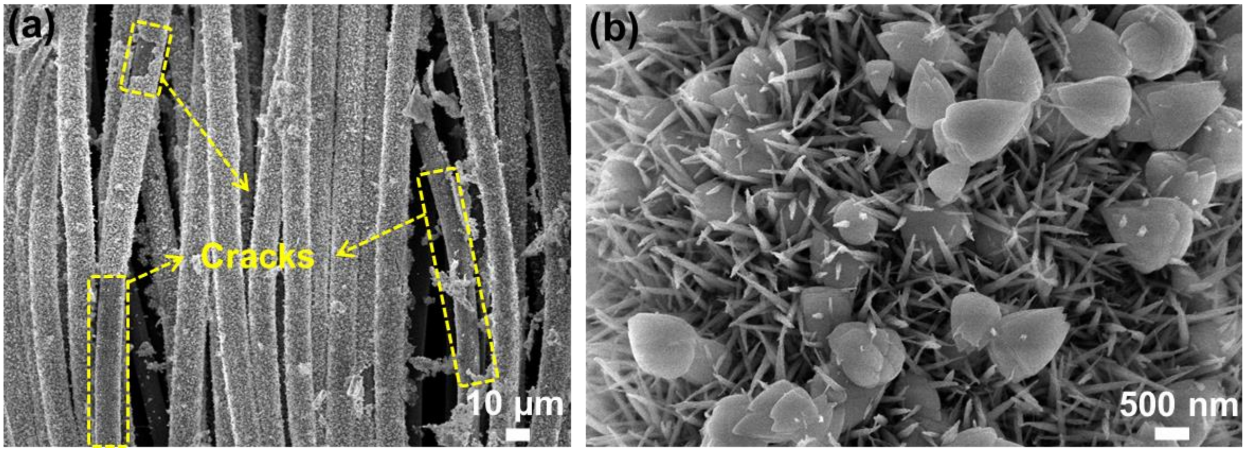

Figure S2. FE-SEM images of the CNP–CP-170 sample at (a) low and (b) high magnifications.

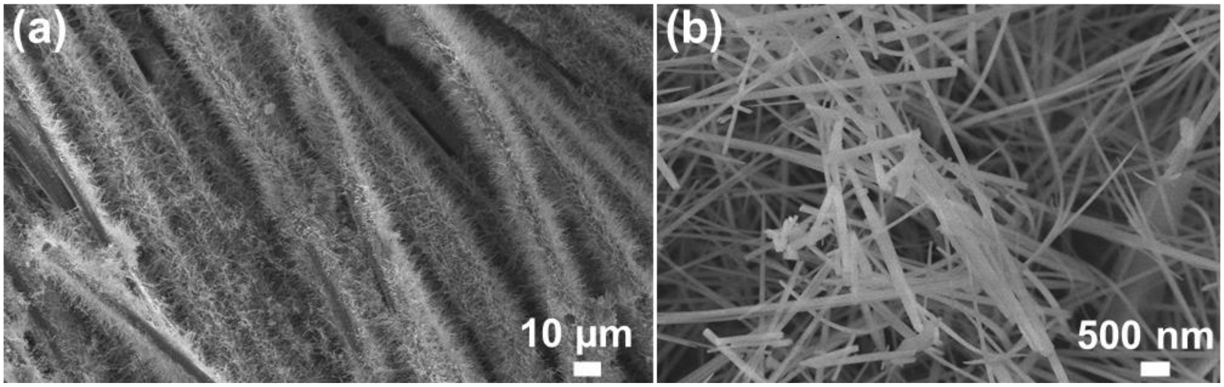

Figure S3. FE-SEM images of the CP-150 sample at (a) low and (b) high magnifications.

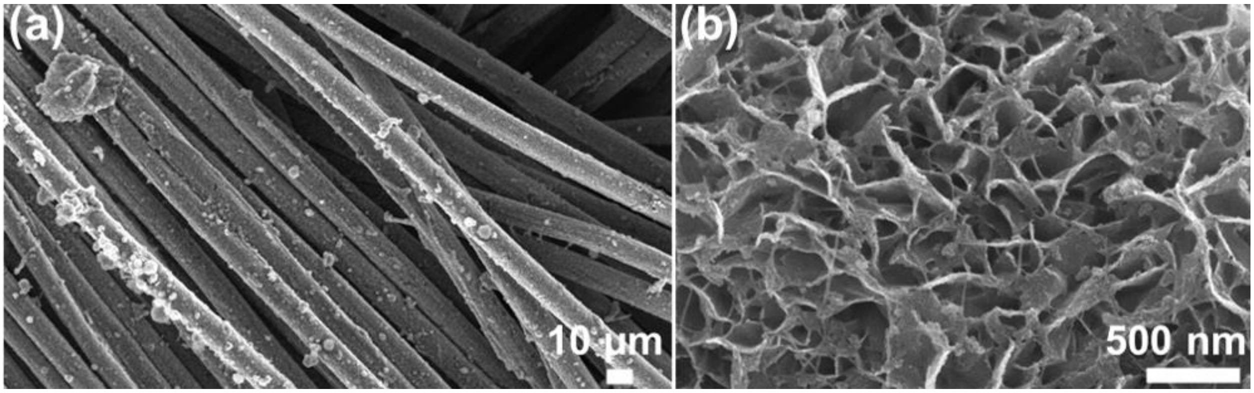

Figure S4. FE-SEM images of the NP-150 sample at (a) low and (b) high magnifications.

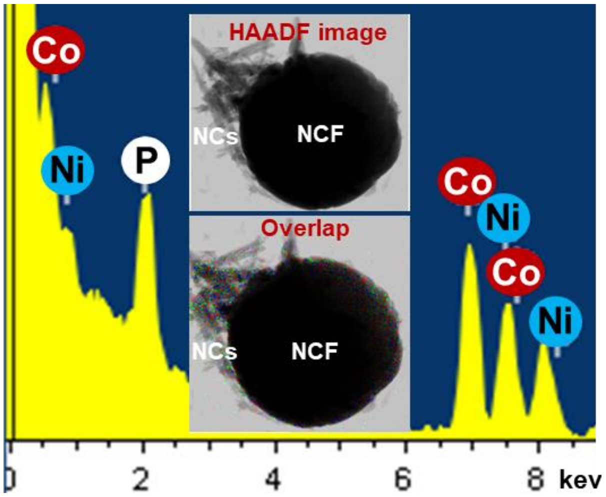

Figure S5. EDS spectrum of the CNP–CP-150 electrode recorded during the TEM measurement. Images placed in the inset are HAADF and overlap images of the hybrid nanoarchitecture.

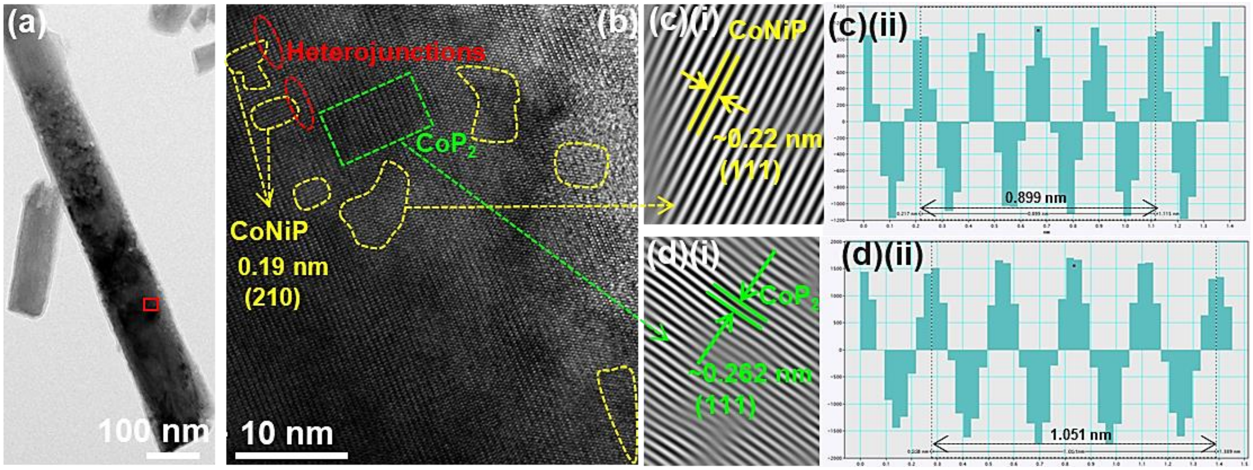

Figure S6. (a) TEM and (b) HR TEM images of the CNP–CP-150 sample. (c)(i) Lattice planes and (c)(ii) lattice distance profiles obtained for the CoNiP phase from the Gatan DigitalMicrograph Software. (d)(i) Lattice planes and (d)(ii) lattice distance profiles obtained for the CoP_2 phase from the Gatan DigitalMicrograph Software.

Note: Figure S6(c)(i) and Figure S6(d)(i) do not represent the single crystalline nature of the prepared material. They seem to be like that since the Gatan DigitalMicrograph Software focused on tiny spots where the lattice planes of the corresponding phase are only presented.

The lattice fringes of the selected area in Figure S6(b) are extracted using the Gatan DigitalMicrograph Software at another spot as indicated in Figure S6(b) to further enquire its phase. From this image, the software calculated the fringe width of ~0.22 nm, which corresponds to the (111) lattice plane of the CoNiP phase. Therefore, it is once more confirmed that the prepared material consists of a dual-phase with a dominant CoP_2 crystalline structure. The same analysis is also performed on the lattice planes related to the CoP_2 at a marked place with green color in Figure S6(b). The resulting image is presented in Figure S6(d)(i). The software estimated the *d*-spacing of ~0.262 nm from Figure S6(d)(ii), which is in good agreement with the value calculated manually in the manuscript.

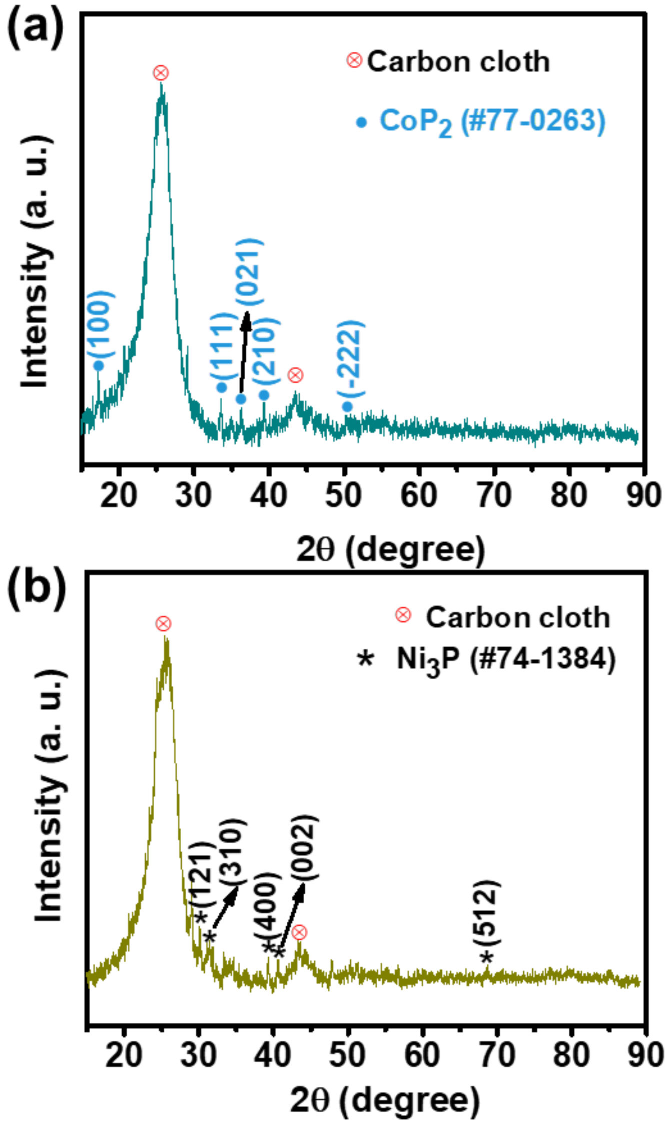

Figure S7. XRD patterns of the (a) CP-150 and (b) NP-150 samples.

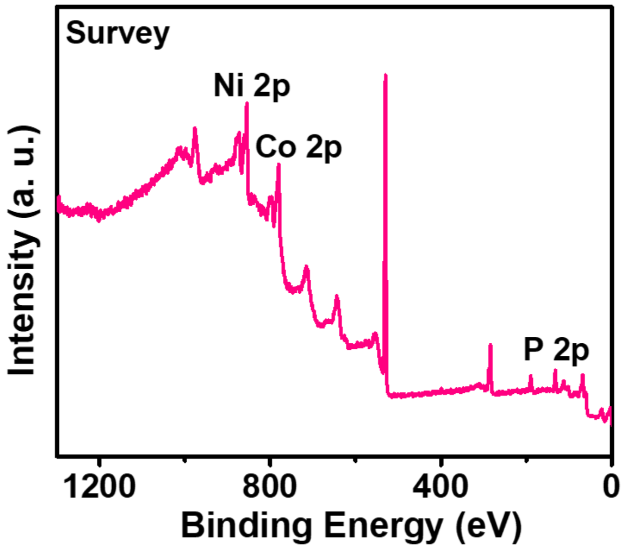

Figure S8. XPS survey scan spectrum of the CNP–CP-150 sample.

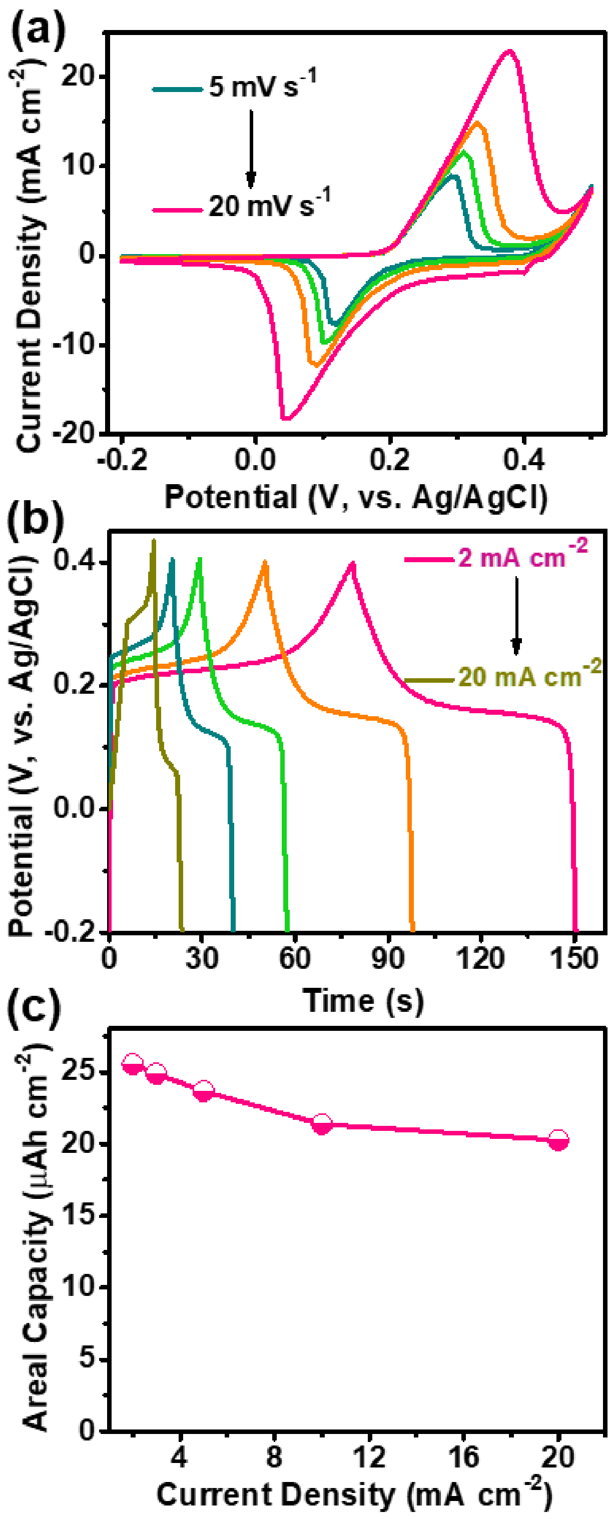

Figure S9. (a) CV curves, (b) GCD curves, and (c) areal capacity values of the CNP–CP-130 electrode.

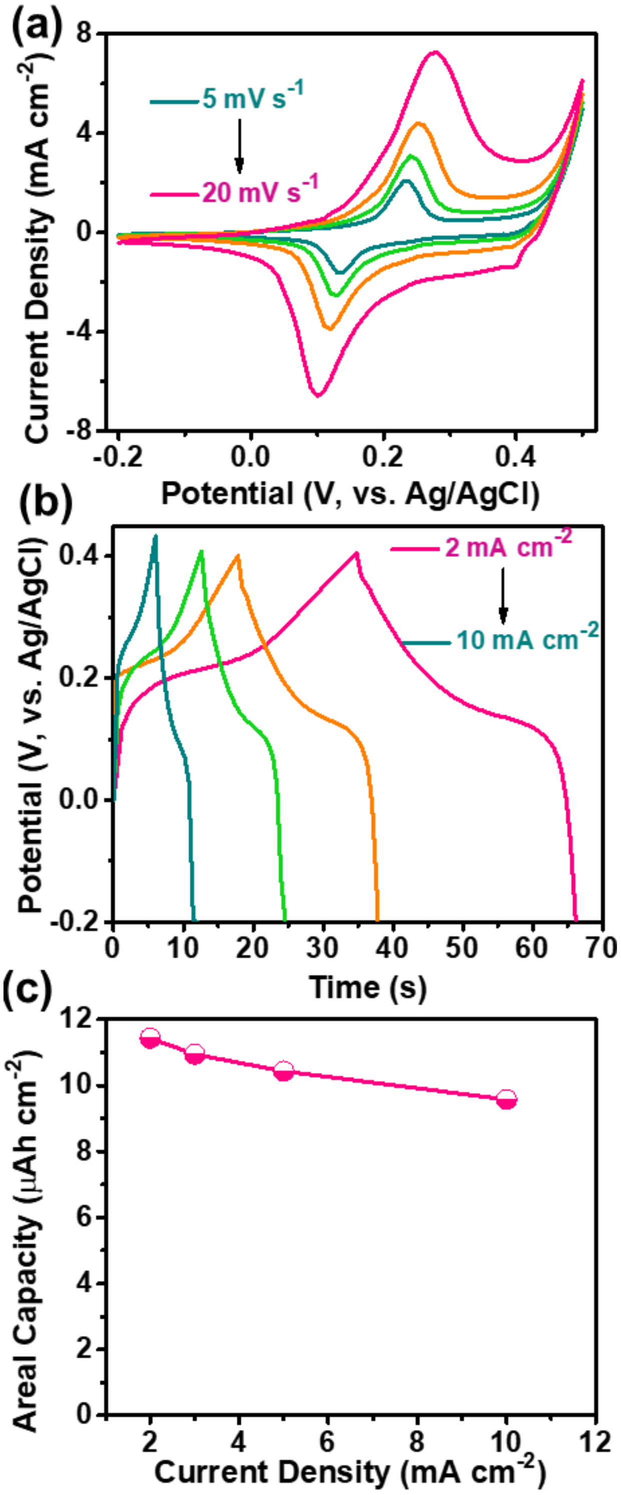

Figure S10. (a) CV curves, (b) GCD curves, and (c) areal capacity values of the CNP–CP-170 electrode.

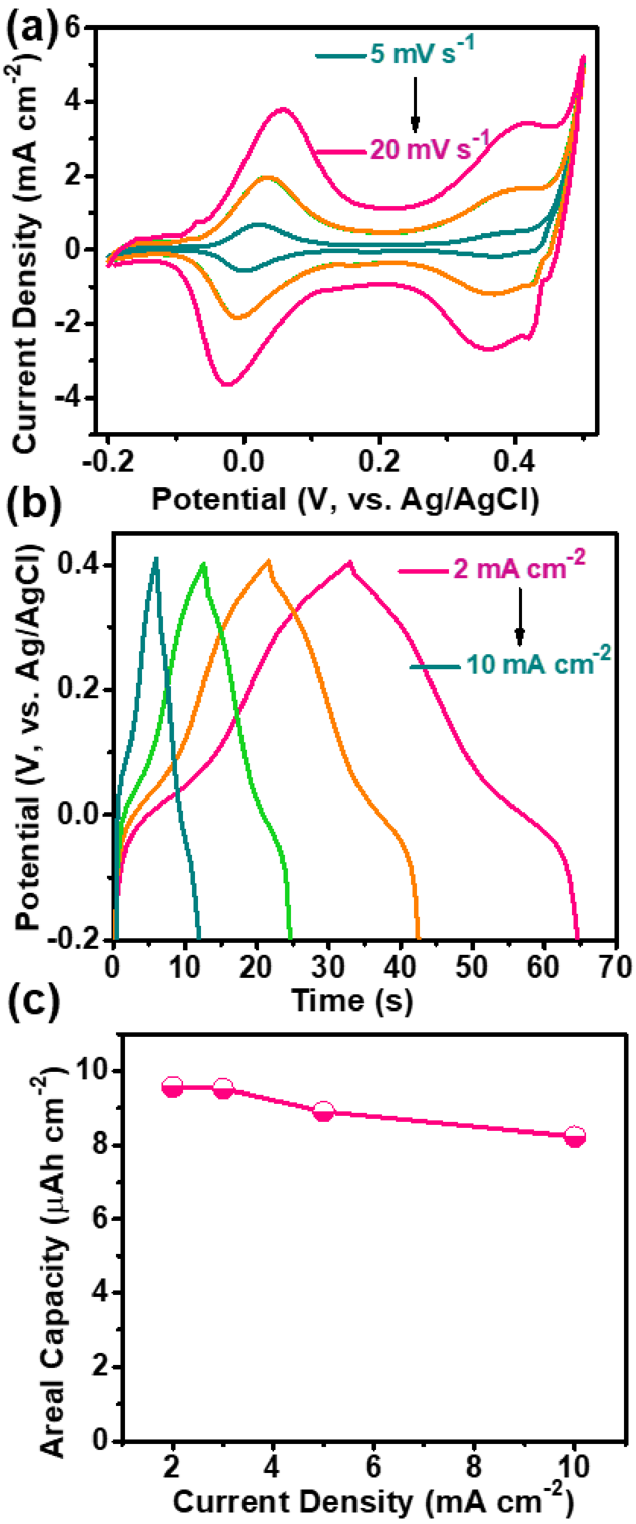

Figure S11. (a) CV curves, (b) GCD curves, and (c) areal capacity values of the CP-150 electrode.

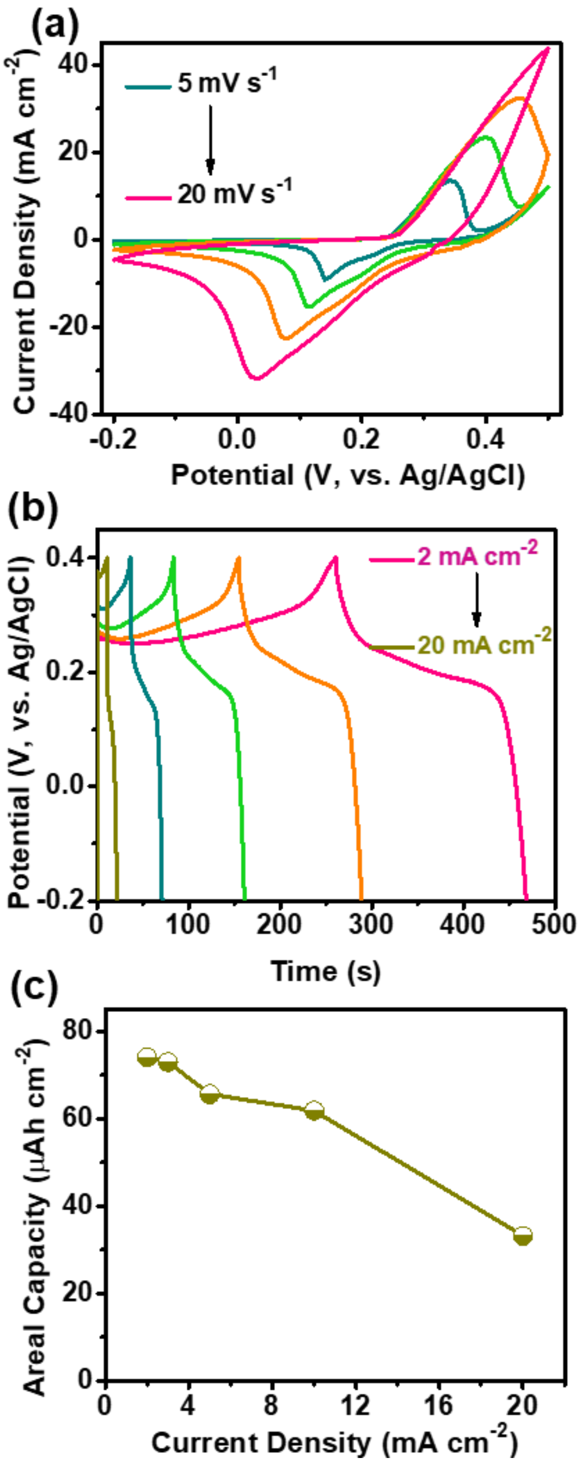

Figure S12. (a) CV curves, (b) GCD curves, and (c) areal capacity values of the NP-150 electrode.

Section-II

Fabrication of negative electrode (AC@CF):

Among several carbon-related materials, activated carbon (AC) was captivated as a promising negative electrode material due to its high porosity, large specific surface area, good electrical conductivity, and high mechanical stability. Therefore, it was chosen as a negative electrode candidate in the present work. The preparation of AC@CF electrode follows as: at first, the AC powder, super P carbon black, and PVDF powders were taken in an agate mortar in the weight ratio of 80:10:10, respectively. This mixture was then ground well for about half an hour. Then, a sufficient volume of NMP solvent was dropped in the above mixture and ground for 10 more minutes. Finally, the viscous slurry was loaded on the cleaned CF substrate within the active area of 1×1 cm^2. After drying at 80 °C overnight, the AC-loaded CF (AC@CF) substrate was pressed at ~3 MPa using a presser instrument for the strong contact of AC material to the CF substrate. The mass of AC material on the CF substrate was noted to be ~3.5 mg cm^{-2}.

The electrochemical properties of the AC@CF electrode were evaluated in a three-terminal system consisting of 2 M KOH electrolyte. The CV profiles of the AC@CF electrode recorded at different sweep rates (Figure S13(a)) exhibited a pseudo-rectangle-like shape, unveiling its non-faradaic-type charge storage mechanism. The nearly linear charge and discharge curves of the AC@CF electrode obtained at different current densities (Figure S13(b)) further endorsed its non-faradaic-type charge storage. At 2 mA cm^{-2}, the AC@CF electrode delivered the areal capacitance of 986 mF cm^{-2}, and it retained 653 mF cm^{-2} even at a high current density of 40 mA cm^{-2}, as shown in Figure S13(c).

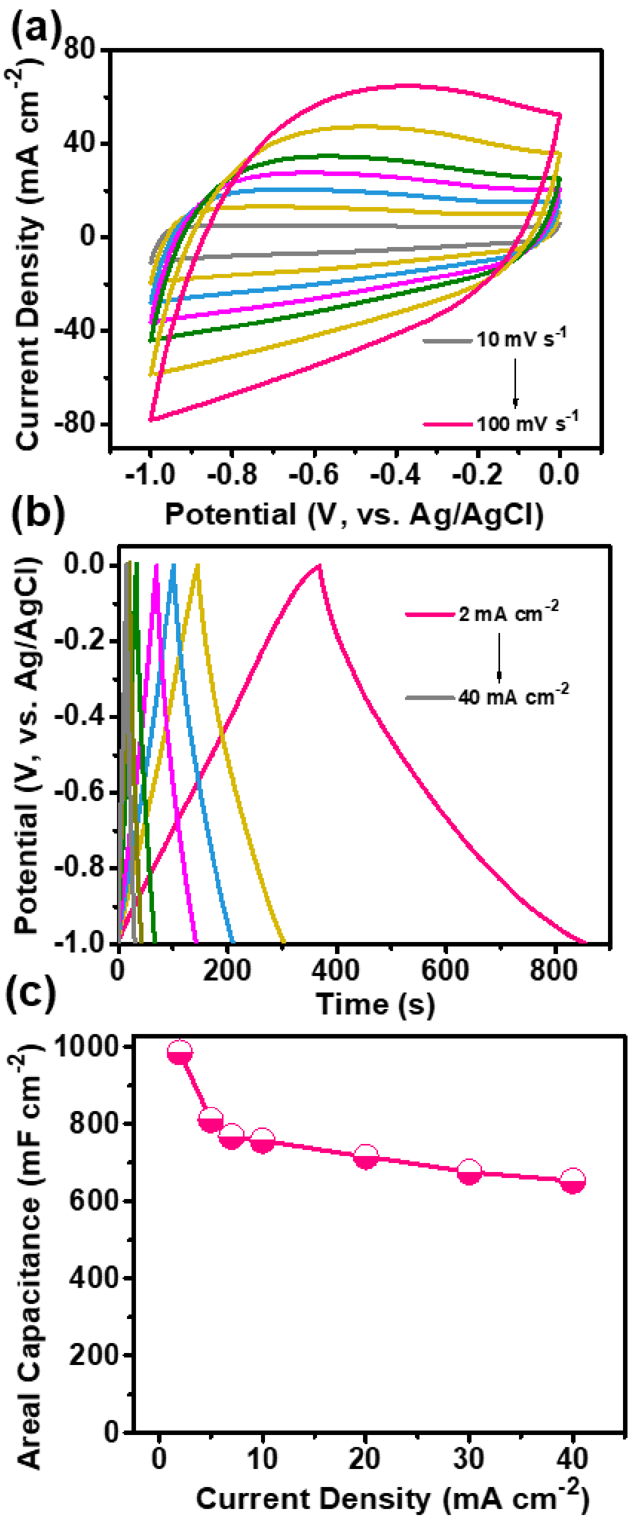

Figure S13. (a) CV curves, (b) GCD curves, and (c) areal capacitance values of the AC@CF electrode.

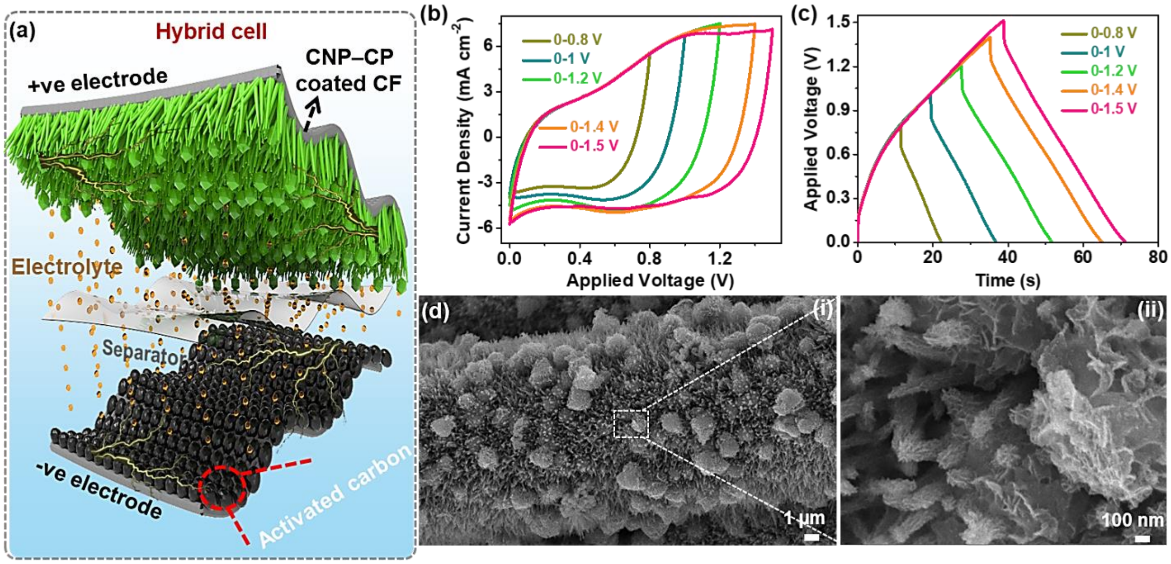

Figure S14. (a) Schematic illustration for the assembling of a hybrid cell with liquid electrolyte. (b) CV and (c) GCD curves of the assembled hybrid cell at different voltage windows. (d)(i) Low- and (d)(ii) high-magnification FE-SEM images of the CNP–CP-150 electrode in the hybrid cell after the durability test.

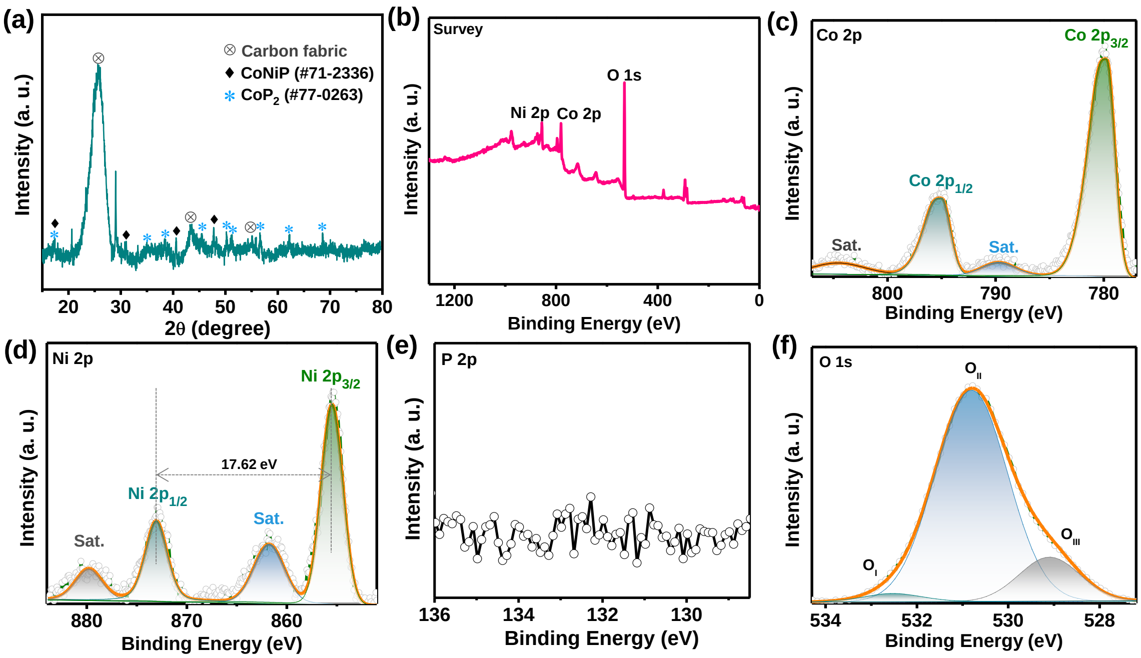

Figure S15. (a) XRD and (b-f) XPS analyses of the CNP–CP-150 electrode in the hybrid cell after the durability test.

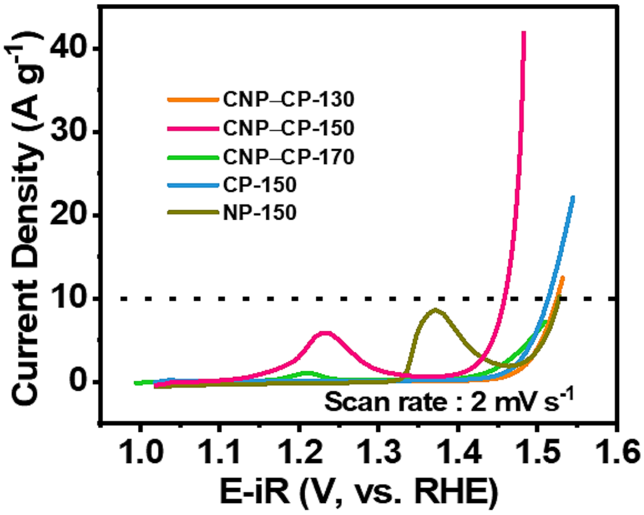

Figure S16. LSV profiles of the CNP–CP-130, CNP–CP-150, CNP–CP-170, CP-150, and NP-150 catalysts, normalized with their respective active mass loadings.

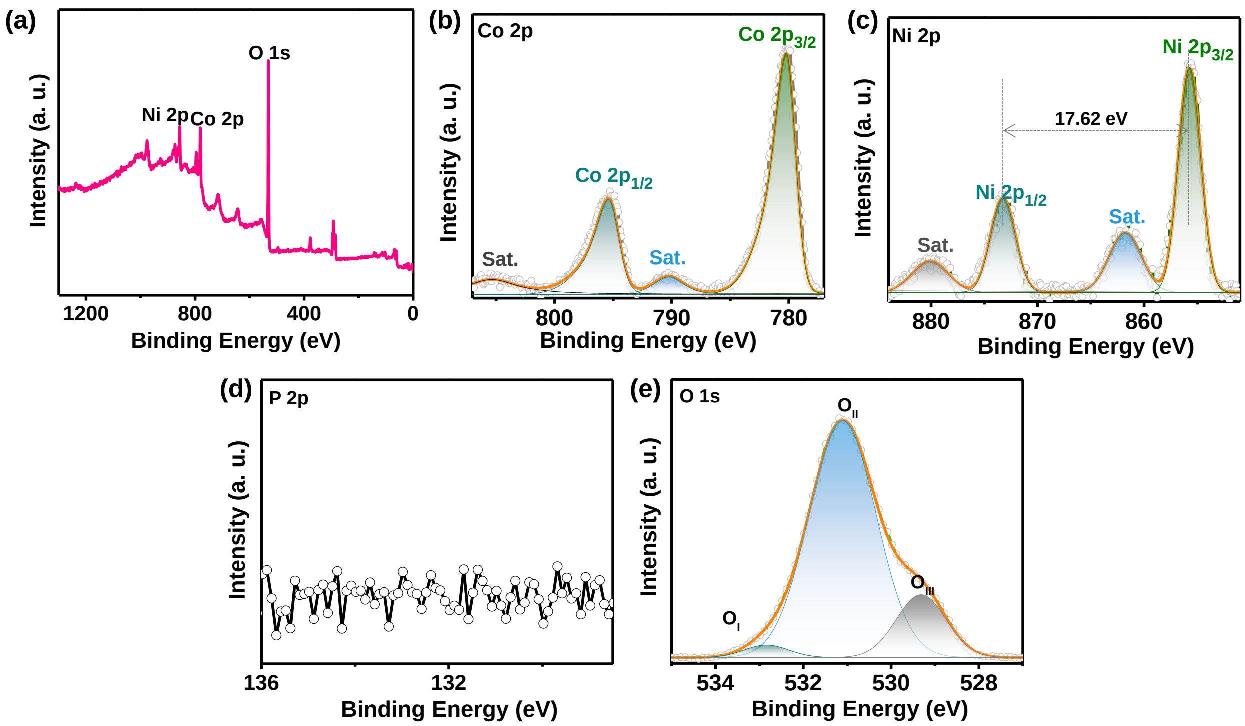

Figure S17. XPS analysis of the CNP–CP-150 catalyst after the OER measurement. (a) XPS survey scan spectrum. HR XPS spectra of (b) Co 2p, (c) Ni 2p, (d) P 2p, and (e) O 1s.

Table S1. EIS parameter values of the CNP–CP-150 electrode measured before and after the durability test

Parameter	Before cycling	After cycling
R_s (Ω cm^{-2})	2.4	2.65
R_{ct} (Ω cm^{-2})	4.7	5.1
CPE (mF)	0.00541	0.00523
W_0 (Ω)	3.2	3.55

Table S2. Comparative energy density values of previously reported literature with our device energy density.

Positive material	Negative material	Electrolyte	Test condition	Energy density	Ref.
CoMn LDH	Activated carbon	1 M LiOH	0.5 A g^{-1}	4.4 Wh kg^{-1}	S[3]
$Mn_3(PO_4)_2$	Activated carbon	6 M KOH	0.5 A g^{-1}	7.6 Wh kg^{-1}	S[4]
$Mn_3(PO_4)_2$	$Mn_3(PO_4)_2$	PVA-Na_2SO_4	0.1 mA cm^{-2}	11.7 Wh kg^{-1}	S[5]
$NiCo_2O_4$–MnO_2	Activated graphene	2 M KOH	0.25 A g^{-1}	9.4 W h kg^{-1}	S[6]
CoP-Ni_2P	ZIF/RGO	1 M KOH	1 mA cm^{-2}	26.9 μWh cm^{-2}	S[7]
CoP-NPC/GS	CoP-NPC	2 M KOH	1 A g^{-1}	10 Wh kg^{-1}	S[8]
D-$NiCo_2O_4$	Activated carbon	PVA-KOH	2 A g^{-1}	9.1 Wh kg^{-1}	S[9]
Mn_3O_4	Graphene	1 M Li_2SO_4	0.05 A g^{-1}	9.6 Wh kg^{-1}	S[10]
$NiCo_2O_4$	$FeSe_2$	PVA/KOH	2.5 mA cm^{-2}	10.4 Wh kg^{-1}	S[11]
$Cu_2P_2O_7$	$Cu_2P_2O_7$	PVA-LiCl	5 A g^{-1}	11.5 Wh kg^{-1}	S[12]
MnO_2/3DGH	V_3S_4/3DGH	PVA/LiCl	4 mA cm^{-2}	7.4 Wh kg^{-1}	S[13]
Ni-Co oxide	Activated carbon	1 M KOH	2 A g^{-1}	12 Wh kg^{-1}	S[14]
**CoNiP–CoP_2**	**Activated carbon**	**2 M KOH**	**2 mA cm^{-2}**	**31 μWh cm^{-2} & 12.4 Wh kg^{-1}**	**This work**

Table S3. Comparative Tafel slope values of the CNP–CP-150 catalyst in comparison with the previously fabricated catalysts.

Active material	Substrate	Electrolyte	Stability test	Tafel slope (mV dec^{-1})	Ref.
$Ni_{1.5}Co_{1.5}P$	Glassy carbon	1 M KOH	12 h	71	S[15]
Ni_xP	Nickel foam	1 M KOH	20 h	63	S[16]
NiCoFeP	Nickel foam	1 M KOH	10 h	45	S[17]
Ar-NiCoP/V	Carbon cloth	1 M KOH	24 h	71	S[18]
NiCoP@Cu_3P	Copper foam	1 M KOH	-	45.6	S[19]
NiCoP	Nickel foam	1 M KOH	28 h	116	S[20]
NiCoP	Nickel foam	1 M KOH	24 h	87	S[21]
NiCoP/C@FeOOH	Glassy carbon	1 M KOH	14 h	69	S[22]
$NiCo_2O_4$/NiCoP	Rotating disk electrode	1 M KOH	12 h	70	S[23]
NiCoP	Nickel foam	1 M KOH	25 h	116	S[24]
**CoNiP–CoP_2**	**Carbon cloth**	**2 M KOH**	**24 h**	**38**	**This work**

References:

S[1] a) H. Liang, C. Xia, Q. Jiang, A. N. Gandi, U. Schwingenschlögl, H. N. Alshareef, *Nano Energy* **2017**, *35*, 331. b) F. Chen, C. Liu, B. Cui, S. Dou, J. Xu, S. Liu, H. Zhang, Y. Deng, Y. Chen, W. Hu, *Journal of Power Sources* **2021**, *482*, 228910. c) X. Yu, B. Lu, Z. Xu, *Advanced Materials* **2014**, *26* (7), 1044. d) S. C. Sekhar, B. Ramulu, S. J. Arbaz, S. K. Hussain, J. S. Yu, *Small Methods* ***2021***, *5*, 2100335.
S[2] G. Nagaraju, S. C. Sekhar, B. Ramulu, J. S. Yu, *Energy Storage Materials* **2021**, *35*, 750.
S[3] A. D. Jagadale, G. Guan, X. Li, X. Du, X. Ma, X. Hao, A. Abudula, *Journal of Power Sources* **2016**, *306*, 526.
S[4] A. A. Mirghni, M. J. Madito, T. M. Masikhwa, K. O. Oyedotun, A. Bello, N. Manyala, *Journal of Colloid and Interface Science* **2017**, *494*, 325.
S[5] P. K. Katkar, S. J. Marje, S. S. Pujari, S. A. Khalate, P. R. Deshmukh, U. M. Patil, *Synthetic Metals* **2020**, *267*, 116446.
S[6] M. Kuang, Z. Q. Wen, X. L. Guo, S. M. Zhang, Y. X. Zhang, *Journal of Power Sources* **2014**, *270*, 426.
S[7] T. Shu, H. Gao, Q. Li, F. Wei, Y. Ren, Z. Sun, J. Qi, Y. Sui, *Nanoscale* **2020**, *12* (40), 20710.
S[8] S. Gayathri, P. Arunkumar, J. H. Han, *Journal of Colloid and Interface Science* **2021**, *582*, 1136.
S[9] V. Veeramani, R. Madhu, S.-M. Chen, M. Sivakumar, *ACS Sustainable Chemistry & Engineering* **2016**, *4* (9).
S[10] Y. Li, X.-M. Li, *RSC Advances* **2013**, *3* (7), 2398.
S[11] C. Ji, F. Liu, L. Xu, S. Yang, *Journal of Materials Chemistry A* **2017**, *5* (11), 5568.
S[12] A. Agarwal, B. R. Sankapal, *Chemical Engineering Journal* **2021**, *422*, 13013.
S[13] T. Zhai, X. Lu, H. Wang, G. Wang, T. Mathis, T. Liu, C. Li, Y. Tong, Y. Li, *Nano Letters* **2015**, *15* (5), 3189.
S[14] C. Tang, Z. Tang, H. Gong, *Journal of The Electrochemical Society* **2012**, *159* (5), A651.
S[15] T. Chen, M. Qian, X. Tong, W. Liao, Y. Fu, H. Dai, Q. Yang, *International Journal of Hydrogen Energy* **2021**, *46* (58), 29889.
S[16] X. Cao, D. Jia, D. Li, L. Cui, J. Liu, *Chemical Engineering Journal* **2018**, *348*, 310.
S[17] Q. Zhang, D. Yan, Z. Nie, X. Qiu, S. Wang, J. Yuan, D. Su, G. Wang, Z. Wu, *ACS Applied Energy Materials* **2018**, *1* (2), 571.
S[18] Y. Yan, J. Lin, J. Cao, S. Guo, X. Zheng, J. Feng, J. Qi, *Journal of Materials Chemistry A* **2019**, *7* (42), 24486.
S[19] X. Ma, Y. Chang, Z. Zhang, J. Tang, *Journal of Materials Chemistry A* **2018**, *6* (5), 2100.
S[20] J. Li, G. Wei, Y. Zhu, Y. Xi, X. Pan, Y. Ji, I. V. Zatovsky, W. Han, *Journal of Materials Chemistry A* **2017**, *5* (28), 14828.
S[21] H. Liang, A. N. Gandi, D. H. Anjum, X. Wang, U. Schwingenschlögl, H. N. Alshareef, *Nano Letters* **2016**, *16* (12), 7718.
S[22] J.-G. Li, Y. Gu, H. Sun, L. Lv, Z. Li, X. Ao, X. Xue, G. Hong, C. Wang, *Nanoscale* **2019**, *11* (42), 19959.
S[23] W. Jin, J. Chen, H. Wu, N. Zang, Q. Li, W. Cai, Z. Wu, *Catalysis Science & Technology* **2020**, *10* (16), 5559.
S[24] S. Surendran, S. Shanmugapriya, P. Zhu, C. Yan, R. H. Vignesh, Y. S. Lee, X. Zhang, R. K. Selvan, *Electrochimica Acta* **2019**, *296*, 1083.
